# Tetrahydromethanopterin as a Two-Carbon Carrier: Formation of *N* ^5^‑Ethyl- and *N* ^5^,*N* ^10^-Ethylene-Tetrahydromethanopterin in *Methanothermobacter marburgensis*

**DOI:** 10.1021/acs.biochem.6c00178

**Published:** 2026-04-29

**Authors:** Maxime G. Laird, Heta Telimaa, Jari Koivisto, Silvan Scheller

**Affiliations:** † Department of Bioproducts and Biosystems, School of Chemical Engineering, 174277 Aalto University, Kemistintie 1, 02150 Espoo, Finland; ‡ Department of Chemistry and Materials Science, School of Chemical Engineering, 174277 Aalto University, Kemistintie 1, 02150 Espoo, Finland

## Abstract

Tetrahydromethanopterin (H_4_MPT) is the main carbon carrier in methane metabolism, which allows the interconversion of a formyl group into a methyl group. Although typically described as a single carbon (C1) carrier, we identified and characterized *N* ^5^-ethyltetrahydromethanopterin (*N* ^5^-ethyl-H_4_MPT) and *N* ^5^,*N* ^10^-(1,1-ethylene)­tetrahydromethanopterin (*N* ^5^,*N* ^10^-ethylene-H_4_MPT) in the model methanogen *Methanothermobacter marburgensis*. The chemical competence of H_4_MPT to accept C2 moieties was assayed through the study of the spontaneous formation of *N* ^5^,*N* ^10^-ethylene-H_4_MPT by the condensation of acetaldehyde with H_4_MPT under standard conditions. The rate constant for this reaction was determined to be 1.53 ± 0.05 M^–1^ s^–1^, and the equilibrium constant was determined to be (8.8 ± 0.5) × 10^3^ M^–1^, which is ca. 35 times higher compared to the analogous reaction with the more common carbon carrier tetrahydrofolate. Biochemical assays with *M. marburgensis* cell lysate suggest that the observed formation of *N* ^5^-ethyl-H_4_MPT relies on the enzymatic reduction of *N* ^5^,*N* ^10^-ethylene-H_4_MPT. These findings illustrate the chemical competency of H_4_MPT to promote biocatalysis with two-carbon moieties and highlight that such reactions might be compatible with established H_4_MPT-mediated microbial pathways.

## Graphical abstract

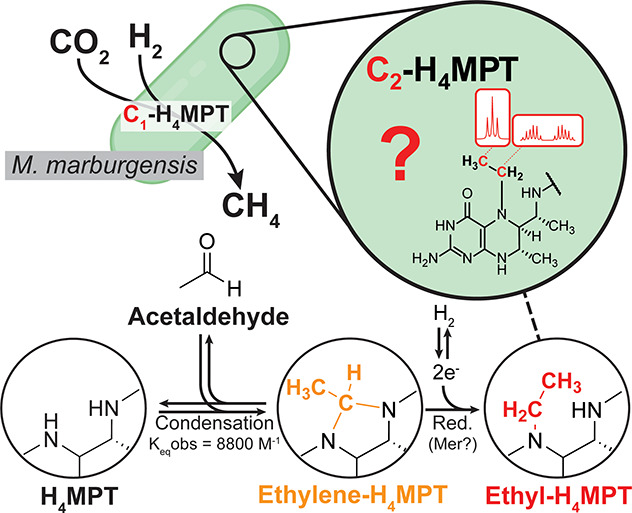

Tetrahydromethanopterin (H_4_MPT) (Figure 1A) is a metabolic one-carbon carrier analog of the more widely utilized tetrahydrofolate (H_4_F) (Figure 1B).^1,2^ H_4_MPT and H_4_F enable enzyme-catalyzed oxidoreductions of the carried carbon between the oxidation states of the formyl and methyl group. Both carriers can bind single carbon units at reactive nitrogen atom 5 (*N* ^5^) that is in conjugation with the pterin ring system and involve the nitrogen atom of the arylamine (*N* ^10^) to form the cyclized intermediate species *N* ^5^,*N* ^10^-methylene and *N* ^5^,*N* ^10^-methenyl. H_4_MPT, however, differs from H_4_F by having a more reactive *N* ^10^ due to the absence of a carbonyl group that is conjugated *para* to the aromatic system (protonated aniline *N* ^10^: p*K*_a_ = +2.4 for H_4_MPT, vs p*K*_a_ = −1.2 for H_4_F).^1^ This higher reactivity contributes to an enhanced stability of the *N* ^5^,*N* ^10^-bound H_4_MPT forms, thus facilitating the cyclization of *N* ^5^-formyl to *N* ^5^,*N* ^10^-methenyl and the oxidation of *N* ^5^-methyl to *N* ^5^,*N* ^10^-methylene groups.

**Figure 1:**
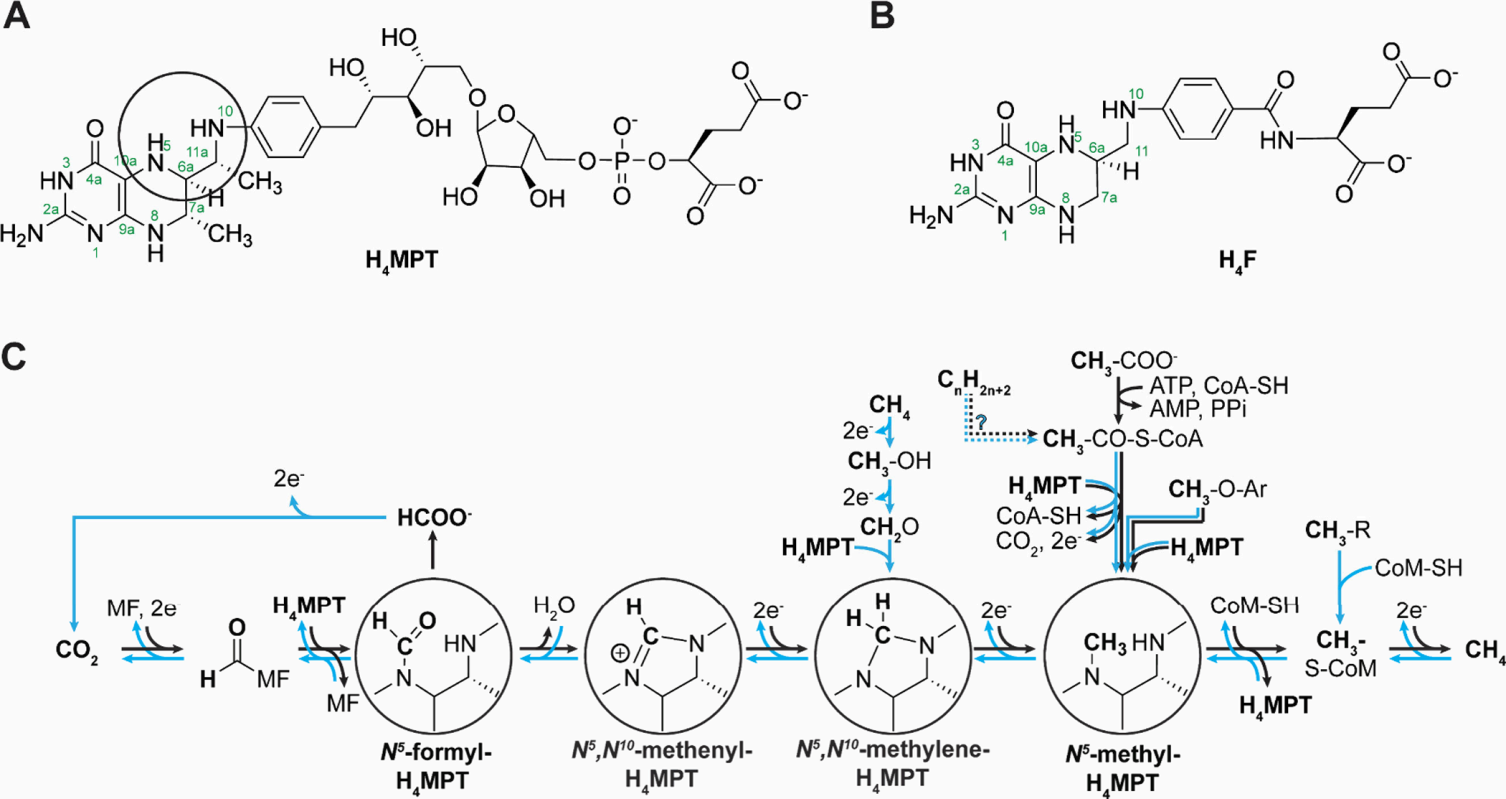
(A) Structure of tetrahydromethanopterin (H_4_MPT) with reactive *N* ^5^ and *N* ^10^ atoms circled. (B) Structure of tetrahydrofolate (H_4_F). (C) Nonexhaustive overview of reported methane-generating and/or CO_2_-generating H_4_MPT-mediated carbon redox catalytic cascades in reductive (black arrows) or oxidative (blue arrows) directions. R = −OH, −SH, −SCH_3_, −NH_2_, −NHCH_3_, and −N­(CH_3_)_2_; MF, methanofuran; CoM, coenzyme M; CoA, coenzyme A; ATP, adenosine triphosphate; AMP, adenosine monophosphate; PPi, pyrophosphate; Ar, aromatic residue.

H_4_MPT is crucial for methane formation in methanogenic archaea and for several metabolic pathways that involve the oxidation of single carbon units to CO_2_ (Figure 1C).^2,3^ Hydrogenotrophic methanogens reduce CO_2_ to methane with H_2_-derived electrons via formyl-, methenyl-, methylene- and methyl-units bonded to H_4_MPT.^3^ Anaerobic oxidation of methane occurs via the same metabolic steps as in hydrogenotrophic methanogenesis but in the reverse direction.^4^ Alternative methanogenic pathways (e.g. methylotrophic, aceticlastic, or methoxydotrophic) can also generate CO_2_ by oxidizing *N* ^5^-methyl-H_4_MPT, following the same metabolic cascade.^3,5^ In aerobic methane oxidation, methane is first oxidized to methanol and then to formaldehyde that condenses with H_4_MPT to *N* ^5^,*N* ^10^-methylene-H_4_MPT for further oxidation steps.^6,7^ In archaeal multicarbon alkane oxidation, the carbon chains are proposed to be split via an unknown pathway into acetyl-CoA units, from which C1 moieties are converted to *N* ^5^-methyl-H_4_MPT and oxidized analogously.^8,9^

Despite H_4_MPT being utilized for diverse metabolisms, this cofactor has been reported exclusively to be a one-carbon carrier. Here, we identified *N* ^5^-ethyl-H_4_MPT and *N* ^5^,*N* ^10^-ethylene-H_4_MPT in the model hydrogenotrophic methanogen *Methanothermobacter marburgensis.* To isolate free H_4_MPT (with no carbon unit bonded to the *N* ^5^ or *N* ^10^) from biomass, the CO_2_/H_2_ growth gas mix was switched to pure H_2_ prior to harvesting to promote the reduction of loaded H_4_MPT (with a carbon bonded to the *N* ^5^ and/or *N* ^10^) toward the release of methane. Unexpectedly, the isolated H_4_MPT pool contained a second pterin species in ratios up to ca. 1:1 relative to free H_4_MPT. ^1^H/^13^C HSQC and HMBC NMR experiments allowed us to identify this additional species to be *N* ^5^-ethyl-H_4_MPT, with the ethyl group bonded to *N* ^5^ (Figure 2). This species is a homologue of the central intermediate *N* ^5^-methyl-H_4_MPT that contains an ethyl instead of a methyl group, which prompted us to investigate whether other C2 homologues of H_4_MPT could also be formed by this microbe. Notably, *N* ^5^,*N* ^10^-ethylene-H_4_MPT (homologue with a 1,1-ethylene group) was detected when the cells were harvested under a conventional CO_2_/H_2_ growth gas mixture (Figure S1).

**Figure 2:**
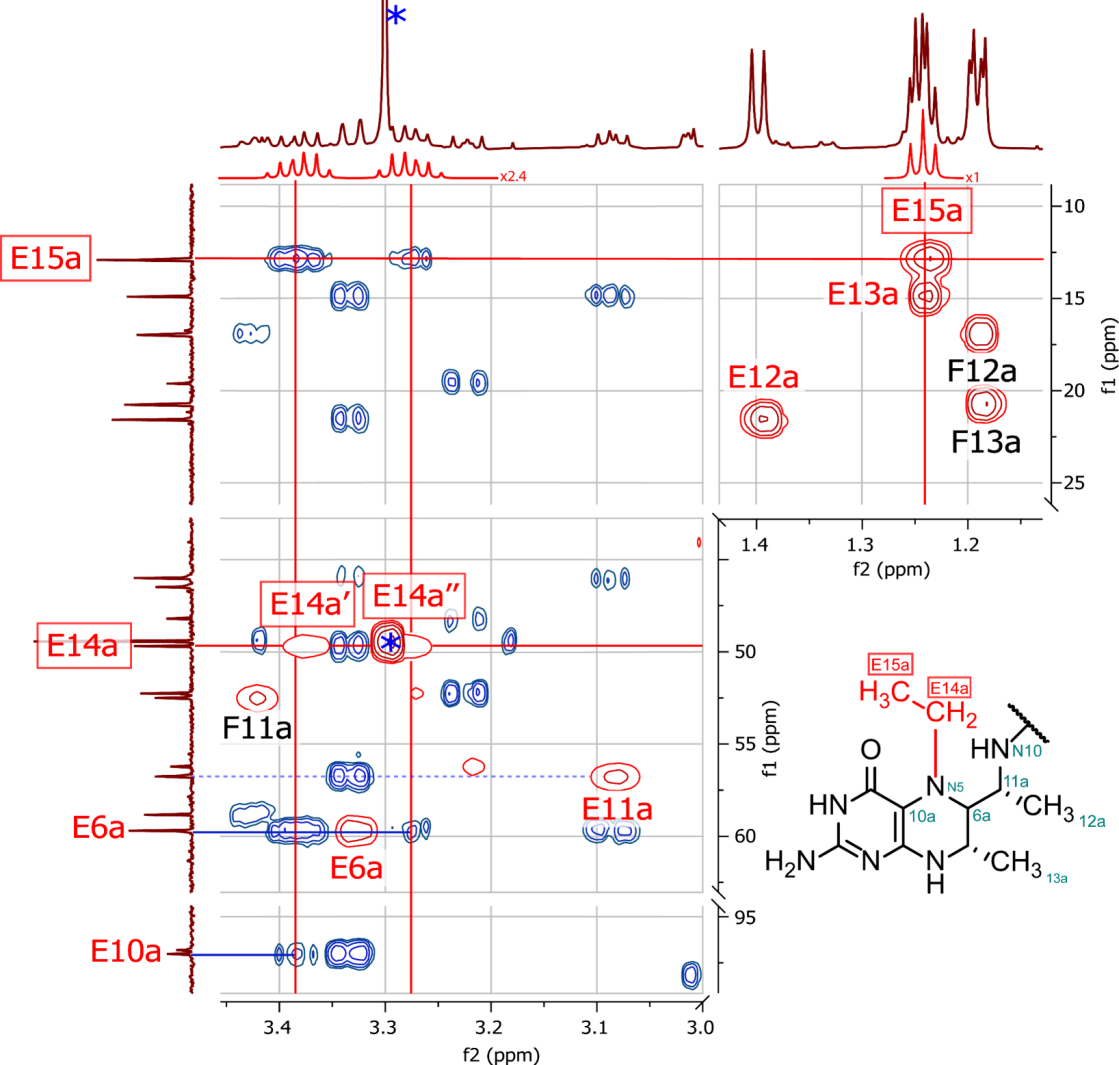
Overlay of 600 MHz ^1^H/^13^C-2D-NMR spectra (f1 = ^13^C, f2 = ^1^H) of a ca. 1:1 mixture of *N* ^5^-ethyl-H_4_MPT and free H_4_MPT. Red contour plot: HSQC. Blue contour plot: HMBC. The ^1^H NMR spectrum of the *N* ^5^-ethyl group is simulated (red horizontal trace) to help identify the signals of the C*H*_3_CH_2_ group that overlap with the methyl groups on the pterins. The signals of the diastereomeric protons of the CH_3_C*H*_2_ group overlap with E6a and with remaining methanol (labeled with *). HSQC signals from *N* ^5^-ethyl-H_4_MPT are labeled with red E, and those from free H_4_MPT, with black F. The identified characteristic signals of the *N* ^5^-ethyl group (boxed red labels, red solid lines) originate from the diastereotopic protons of the methylene group (−*CH*_2_CH_3_, E14A′ and E14A″) and from the terminal methyl group (−CH_2_ *CH*_3_, E15A). The bonding of the ethyl group to the *N* ^5^ is deduced from the HMBC cross-peaks (solid blue lines) of both protons of the CH_3_C*H*_2_ group with carbon 6 (^3^ *J*_C,H_) and of the downfield proton of the CH_3_C*H*_2_ group with carbon 10 (^3^ *J*_C,H_). No cross-peaks between the signals of the CH_3_C*H*_2_ group to those of carbon 11 are observed, which would be a ^4^ *J*_C,H_ (blue dotted line). The ethyl group cannot be bonded to *N* ^10^, because the observed cross-peak between the signals of the CH_3_C*H*_2_ group and those of carbon 10 would be a visible ^6^ *J*_C,H_ coupling, while a ^3^ *J*_C,H_ coupling to carbon 11 is absent (see the Supporting Information, section I-1).

To confirm the identity of *N* ^5^,*N* ^10^-ethylene-H_4_MPT and *N* ^5^-ethyl-H_4_MPT, both species were synthesized from free H_4_MPT. *N* ^5^,*N* ^10^-Ethylene-H_4_MPT is expected to be accessible via acetaldehyde condensation with H_4_MPT, analogously to the reported synthesis of *N* ^5^,*N* ^10^-ethylene-H_4_F from H_4_F.^10^ The condensation of purified H_4_MPT with different acetaldehyde concentrations was followed via UV spectroscopy under standard conditions (pH = 7.0 and *T* = 25 °C) and the dissociation of *N* ^5^,*N* ^10^-ethylene-H_4_MPT to acetaldehyde and free H_4_MPT via *in situ* ^1^H NMR spectroscopy (Figure S2). The kinetic parameters of the reaction (Table 1) were quantified by fitting time-dependent concentration data of the individual species to a reaction model. Considering free acetaldehyde (and not the sum with hydrated forms), the second-order kinetic constant for the condensation (*k*_C,obs_) and the first-order kinetic constant for the hydrolysis (*k*_H,obs_) were determined as *k*_H,obs_ = (1.75 ± 0.11) × 10^–4^ s^–1^ and *k*_C,obs_ = 1.53 ± 0.05 M^–1^·s^–1^, respectively. The hydrolysis reaction monitoring via ^1^H NMR spectroscopy allowed simultaneous quantification of acetaldehyde and hydrated acetaldehyde along with free H_4_MPT and *N* ^5^,*N* ^10^-ethylene-H_4_MPT. Therefore, the equilibrium constant of the reaction was determined to be *K*_obs_ = (8.8 ± 0.5) × 10^3^ M^–1^. This value is approximately 35-fold higher than the reported value for the analogous reaction with H_4_F^10^ (*K*_obs_ = 249 M^–1^, calculated from the published value of *K*_obs_ = 121 ± 2 M^–1^ that is based on total acetaldehyde concentration, considering the hydration constant of acetaldehyde,^11^ *K*_hydration_ = 1.06, and neglecting acetaldehyde oligomers). The stronger acetaldehyde-binding capabilities of H_4_MPT compared to H_4_F are consistent with the higher reactivity of its *N* ^10^ that stabilizes the condensed form,^1^ making H_4_MPT a more suitable cofactor for the processing of an ethylene group (experimental details in Supporting Information, section II-14 to II-16).

**Table 1:** Kinetic and Thermodynamic Constants for Acetaldehyde Condensation with H_4_MPT

Constant		Value ± CI 95%
Condensation rate constant	*k*_C,obs_	1.53 ± 0.05 M^–1^·s^–1^
Hydrolysis rate constant	*k*_H,obs_	(1.75 ± 0.11) × 10^–4^ s^–1^
Equilibrium constant	*K*_C,obs_	(8.8 ± 0.5) × 10^3^ M^–1^
Gibbs free energy	Δ*G*°′_C_	–22.51 ± 0.13 kJ·mol^–1^

*N* ^5^-Ethyl-H_4_MPT was synthesized by potassium borohydride reduction of *N* ^5^,*N* ^10^ *-*ethylene-H_4_MPT, analogous to the reported method for the synthesis of C1 H_4_MPT species.^12^ Both species, alongside their C1 homologues, were characterized via UV and ^1^H NMR spectroscopy at pH 7.0. The resulting library of H_4_MPT species allows their quantification in complex mixtures by integrating the ^1^H NMR signals from their aniline rings (2b/6b and 3b/5b) and those from the structural methyl groups of their pterin moiety (12a and 13a) (Figure 3).

**Figure 3:**
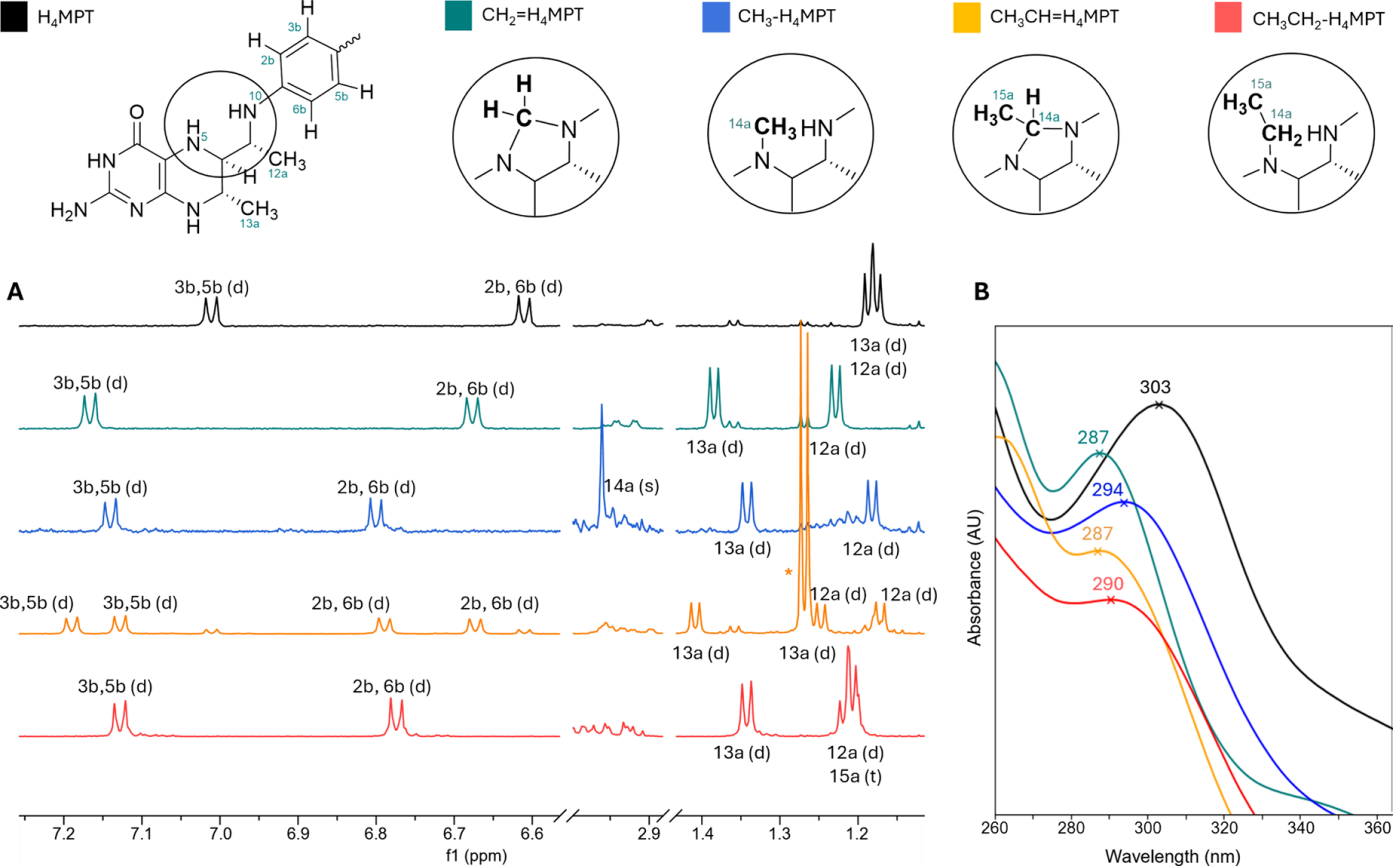
600 MHz ^1^H NMR and UV spectroscopic characterization of H_4_MPT (black) and its methylene (teal), methyl (blue), ethylene (orange), and ethyl (red) derivatives at pH = 7.0. (A) Characteristic ^1^H NMR signals of aromatic protons (3b/5b, 2b/6b) and structural methyl groups (12a/13a). To distinguish between the methyl and ethyl derivatives, the 14a-methyl signal at 2.96 ppm is characteristic of *N* ^5^-methyl-H_4_MPT, and the 15a-C*H*_3_CH_2_ signal at 1.21 ppm that overlaps with 12a is characteristic of *N* ^5^-ethyl-H_4_MPT (see the Supporting Information, section I-1 and Figure S3). For *N* ^5^,*N* ^10^-ethylene-H_4_MPT, both diastereoisomers (stereochemistry at CH_3_ *C*H= of the ethylene group) are present in a ratio of ca. 50:50. C*H*_3_CH= groups are located at 0.89 and 1.47 ppm. One CH_3_C*H*= group is at 5.41 ppm, and the other one overlaps with the water signal (see the Supporting Information, section I-2 and Figure S4). *Excess acetaldehyde hydrate utilized to generate *N* ^5^,*N* ^10^-ethylene-H_4_MPT overlaps with 13a. (B) UV profiles of each H_4_MPT species and their λ_max_ values. The measured absorbances at λ_max_ range from 0.4 to 0.9 AU (Figure S5). The spectra were normalized at their respective λ_max_ value to free H_4_MPT and staggered by −0.05 AU intervals for better visualization.

To study the requirements for the formation of *N* ^5^ *-*ethyl-H_4_MPT in *M. marburgensis*, we carried out *in vitro* experiments with lysates of *M. marburgensis* cells that were harvested under the conventional CO_2_/H_2_ growth gas mixture from bioreactor cultures. The cell lysates were sparged with either pure H_2_ or pure N_2_, and the pterin pool was isolated. ^1^H NMR analysis of the resulting fractions shows that H_2_ treatment led to >75% *N* ^5^-ethyl-H_4_MPT, whereas untreated and N_2_-treated cell lysate extracts were found to contain less than 10% *N* ^5^-ethyl-H_4_MPT (Figure 4A). These *in vitro* assays align with the high level of *N* ^5^-ethyl-H_4_MPT that was observed *in vivo* when cells were treated with H_2_ in the absence of CO_2_ before harvest. The hydrogen requirement for both *in vivo* and *in vitro* formation of *N* ^5^-ethyl-H_4_MPT suggests that the ethylation of H_4_MPT requires H_2_-derived reducing equivalents in addition to an endogenous two-carbon donor.

**Figure 4:**
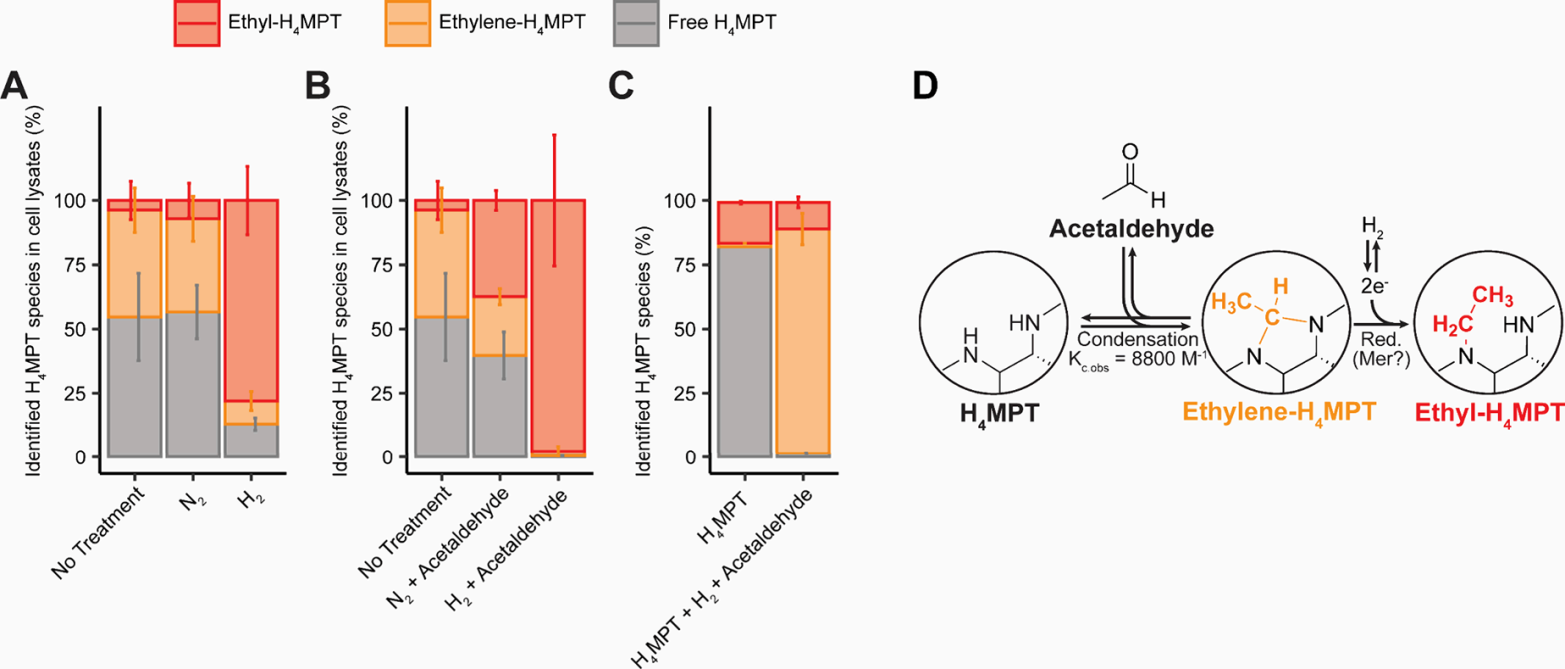
Formation of *N* ^5^-ethyl-H_4_MPT and *N* ^5^,*N* ^10^-ethylene-H_4_MPT in cell lysates of *M. marburgensis*. (A) Influence of H_2_ on the H_4_MPT pool. The relative fractions of H_4_MPT, ethyl-H_4_MPT and ethylene-H_4_MPT in the purified pterin pool were quantified via ^1^H NMR spectroscopy after different treatments. No treatment corresponds to a 3 s flushing with N_2_. Treatments included 90 min of flushing with either N_2_ or H_2_. Prior to purification, all samples were incubated for 1.5 h at 60 °C, which is close to the optimal growth temperature for *M. marburgensis*. (B) Same assays but with the addition of 3 mM acetaldehyde after gassing in order to maximize the amount of *in situ*-produced *N* ^5^,*N* ^10^-ethylene-H_4_MPT. (C) Cell lysate-free assay: extracted H_4_MPT containing minor *N* ^5^-ethyl-H_4_MPT vs reaction mixture 25 mM acetaldehyde, 0.25 mM H_4_MPT, 100% H_2_. Inter- and intraexperiment statistical comparative *p*-values are available in Figure S6. (D) Putative *in vitro* reaction cascade between acetaldehyde and *N* ^5^-ethyl-H_4_MPT.

To increase the fraction of H_4_MPT species that contain a two-carbon moiety in our *in vitro* cell lysate experiments, acetaldehyde was added as a two-carbon donor after gas treatment. The combination of acetaldehyde and H_2_ treatment resulted in *N* ^5^-ethyl-H_4_MPT being the most abundant species in the isolated pterin pools (Figure 4B). Adding acetaldehyde in N_2_-gassed extracts also increased the proportion of isolated *N* ^5^-ethyl-H_4_MPT, suggesting that the cells contain reducing equivalents for the *in vivo* formation of *N* ^5^-ethyl-H_4_MPT even without additional H_2_ treatment. Conversion of acetaldehyde to the ethyl group of *N* ^5^-ethyl-H_4_MPT was confirmed by analogous cell lysate experiments with ^13^C_2_-acetaldehyde and H_2_ treatment, where the resulting isolated pterin pools showed comparable proportions of *N* ^5^-ethyl-H_4_MPT but with a ^13^C_2_-labeled ethyl group, as confirmed via HSQC NMR spectroscopy (Figure S7). The requirement of a cell lysate for this reaction was demonstrated by the absence of spontaneous formation of *N* ^5^-ethyl-H_4_MPT from acetaldehyde and free H_4_MPT under a H_2_ atmosphere (Figure 4C).

Our results highlight that (1) cell-lysate-promoted formation of *N* ^5^-ethyl-H_4_MPT relies on reducing equivalents, (2) acetaldehyde can act as a C2 moiety donor in this reaction, and (3) acetaldehyde can condense with free H_4_MPT to form *N* ^5^,*N* ^10^-ethylene-H_4_MPT under physiological conditions. These three findings support the hypothesis that *N* ^5^-ethyl-H_4_MPT is formed by the reduction of *N* ^5^,*N* ^10^-ethylene-H_4_MPT. This reaction would be analogous to the metabolic step catalyzed by the enzyme methylene-H_4_MPT reductase (Mer) but proceeding with C2 units on H_4_MPT instead of C1 units (Figure 4D). The Mer reaction is known to acquire the reducing equivalents ultimately from hydrogen in hydrogenotrophic methanogens. To confirm that *N* ^5^-ethyl-H_4_MPT formation is catalyzed by a substrate-promiscuous Mer, additional experiments with the purified enzyme would be required.

The two-carbon units of *N* ^5^-ethyl-H_4_MPT and *N* ^5^,*N* ^10^-ethylene-H_4_MPT detected in cell-lysates of *M. marburgensis* are likely formed through carbon salvage pathways and accumulate under our bioreactor conditions as dead-end metabolites. Acetaldehyde produced by the oxidative degradation of H_4_MPT itself in methanogens^13^ could be a potential source of C2 moieties. Endogenous acetaldehyde could also be produced from the decarboxylation of cellular pyruvate, which has been described in the anaerobic archaeon *Pyrococcus furiosus* to be catalyzed as a side-reaction of the enzyme pyruvate:ferredoxin oxidoreductase (Por), particularly under CO_2_ limiting conditions.^14^ Por is common in anaerobic microbes,^15^ and a homologue is also present in *M. marburgensis*.^16^ The identification of endogenous two-carbon donors with our experimental approach would require a catalytically active cell lysate without two-carbon donors.

In conclusion, we show that H_4_MPT spontaneously condenses with acetaldehyde to a C2-loaded form that is ca. 35 times more stable compared to the H_4_F analog and that C2-H_4_MPT derivatives can be formed via intracellular processes. These findings illustrate not only the chemical competency of H_4_MPT for two-carbon reactions but also the putative compatibility of such reactions with established H_4_MPT-mediated microbial pathways. Consequently, the pool of metabolites and enzymatic reactions to be considered in biological systems needs to be extended from established C1 units to C2 units. Derivatives of H_4_MPT that carry multicarbon units may serve as substrates for synthetic biocatalytic applications, and they may have already been utilized by nature for the catalysis of non-C1 H_4_MPT-mediated redox reactions. It is tempting to speculate that the unknown catabolic steps in archaeal ethane oxidation^8^ rely on the reaction cascade from *N* ^5^-ethyl-H_4_MPT to acetaldehyde to reroute an ethyl group into established pathways.

## Supplementary Material



## Notes

Method section, additional data for UV characterization of H_4_MPT derivatives, plots of kinetic model fitting to experimental data for condensation and hydrolysis experiments, cell lysate experiments statistic data, NMR and MS characterization data for free H_4_MPT/*N* ^5^-ethyl-H_4_MPT mixture and *N* ^5^,*N* ^10^-ethylene-H_4_MPT (PDF)
