# Supplementary material for: Tetrahydromethanopterin as a Two-Carbon Carrier: Formation of N 5‑Ethyl- and N 5,N 10-Ethylene-Tetrahydromethanopterin in Methanothermobacter marburgensis
Source: Biochemistry. 2026 Apr 29;65(10):1652–7. doi: 10.1021/acs.biochem.6c00178 (PMC13192316; doi:10.1021/acs.biochem.6c00178)
Supplement: Supplementary file 1 [file bi6c00178_si_001.pdf]

SUPPORTING INFORMATION – METHODS AND ADDITIONAL FIGURES

TETRAHYDROMETHANOPTERIN AS A TWO-CARBON CARRIER: FORMATION OF *N*^{5}-ETHYL- AND *N*^{5},*N*^{10}-ETHYLENE-TETRAHYDROMETHANOPTERIN IN METHANOTHERMOBACTER MARBURGENSIS

Maxime G. Laird[a&], Heta Telimaa[a&], Jari Koivisto[b], Silvan Scheller[a*]

a M. G. Laird, H. Telimaa, Prof. Dr. S. Scheller*
Department of Bioproducts and Biosystems
School of Chemical Engineering, Aalto University
Kemistintie 1, 02150 Espoo, Finland
E-mail: silvan.scheller@aalto.fi

b Dr. J. Koivisto
Department of Chemistry and Materials Science
School of Chemical Engineering, Aalto University
Kemistintie 1, 02150 Espoo, Finland

& Both authors contributed equally to this work

TABLE OF CONTENTS

I – SUPPLEMENTARY FIGURES AND TABLES S3
Figure S1: Identification of *N*^{5},*N*^{10}-ethylene-H_4MPT in cell lysates S3
Figure S2: Time courses of kinetic experiment for the condensation of acetaldehyde to H_4MPT and directly following the de-condensation S4
Figure S3: Assignment table for ^{1}H- and ^{13}C-NMR signals shifts of H_4MPT and *N*^{5}-ethyl-H_4MPT S6
Figure S4: Assignment table for ^{1}H- and ^{13}C-NMR signals shifts of of *N*^{5},*N*^{10}-ethylene-H_4MPT diastereoisomers S7
Figure S5: UV absorbance values at λ_{max} for H4MPT derivatives S8
Figure S6: Statistical analysis of *in vitro* cell lysate experiments S8
Figure S7: HSQC-spectrum of *N*^{5}-$^{13}C_2$-ethyl-H_4MPT from cell lysates assays with $^{13}C_2$-acetaldehyde S9
1 – Characterization data for H_4MPT and *N*^{5}-ethyl-H_4MPT in a ca. 1:1 mixture S10
Figure S8 : Structure and atom labelling of H_4MPT used for spectra analysis. S10
Figure S9: Structure and atom labelling of *N*^{5}-ethyl- H_4MPT used for spectra analysis. S10
Figure S10: 600 MHz ^{1}H-NMR spectrum with assignments S11
Figure S11: ^{1}H-NMR Simulation of the ethyl group of *N*^{5}-ethyl-H_4MPT and overlap with ^{1}H-NMR spectrum S12
Figure S12: 150 MHz ^{13}C-NMR spectrum with assignments S13
Figure S13: HSQC and HMBC spectra overlays S14
Figure S14: Assignment table for HSQC spectrum S15
Figure S15: MS-QTOF spectrum S16
2 – Characterization data for *N*^{5},*N*^{10}-ethylene-H_4MPT diastereoisomers S17
Figure S16: Structure and atom labelling of *N*^{5},*N*^{10}-ethylene-H_4MPT (isomers A and B) used for spectra analysis. S17
Figure S17: 600 MHz ^{1}H-NMR spectrum S18
Figure S18: HSQC and HMBC spectra (whole spectrum) S19
Figure S19: HSQC and HMBC spectra (expansion) S20
Figure S20: Assignment table for HSQC NMR spectrum S21
Figure S21: Assignment table for HMBC NMR spectrum S21
Figure S22: EASY-ROESY NMR spectrum S22
Figure S23: MS-QTOF spectrum S23

II – MATERIALS AND METHODS S24
1 – Methodology for anaerobic conditions S24
2 – Cultivation of Methanothermobacter marburgensis S24
3 – Cell harvesting S24
4 – Cell lysate preparations S25
5 – Purification of H_4MPT and *N*[5]-Ethyl-H_4MPT from *M. marburgensis* biomass S25
6 – Pterin desalting/concentration S26
7 – Synthesis of the C1-carrying H_4MPT forms (*N*[5],*N*[10]-methylene- and *N*[5]-methyl-) from free H_4MPT S26
8 – Synthesis of the C2-carrying H_4MPT forms (*N*[5],*N*[10]-ethylene-, *N*[5]-ethyl-) from free H_4MPT: S27
9 – *In vitro* cell lysate experiments S27
10 – Spontaneous *N*[5],*N*[10]-ethylene-H_4MPT reduction assay S27
11 – Single timepoint NMR data acquisition (cell lysate assay, spontaneous *N*[5],*N*[10]-ethylene-H_4MPT reduction assay, pterin characterization) S27
12 – NMR data processing for relative quantification (*in vitro* cell lysate experiments, spontaneous *N*[5],*N*[10]-ethylene-H_4MPT reduction assay) S28
13 – NMR spectra simulation S29
14 – Quantification of free H_4MPT and ethylene-H_4MPT for kinetic studies (acetaldehyde condensation) in UV-Vis S29
15 – Quantification of free H_4MPT and *N*[5],*N*[10]-ethylene-H_4MPT for kinetic studies (*N*[5],*N*[10]-ethylene-H_4MPT hydrolysis) through multiple timepoint NMR spectra acquisition S30
16 – Kinetic modelling of acetaldehyde condensation with free H_4MPT and *N*[5],*N*[10]-ethylene-H_4MPT hydrolysis reactions: rate and equilibrium constants determination (CoPaSi) S30
17 – MS-QTOF analysis S31

III – LITERATURE FOR SUPPORTING INFORMATION S32

I – SUPPLEMENTARY FIGURES AND TABLES

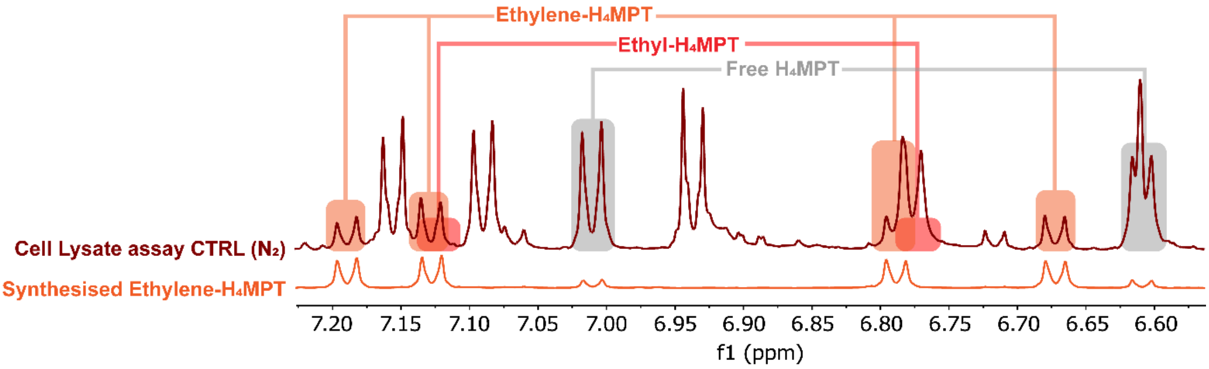

Figure S1: Identification of *N*^{5},*N*^{10}-ethylene-H_4MPT in cell lysates

Top: Expansion of the 600 MHz ^{1}H-NMR spectrum of the concentrated pterin pool isolated from *M. marburgensis cells.* The cells were harvested under the standard growth conditions without any addition or gas treatment. *N*^{5},*N*^{10}-ethylene-H_4MPT gives rise to two pairs of doublets with the same intensities in the aromatic region (signals 2b/6b and 3b/5b, see Figure S4), due to the presence of two diastereoisomers at ca. 1:1 ratio. Bottom: Comparison with the ^{1}H-NMR spectrum of synthesized *N*^{5},*N*^{10}-ethylene-H_4MPT. For full NMR characterization and MS spectrum of *N*^{5},*N*^{10}-ethylene-H_4MPT, see section I-2.

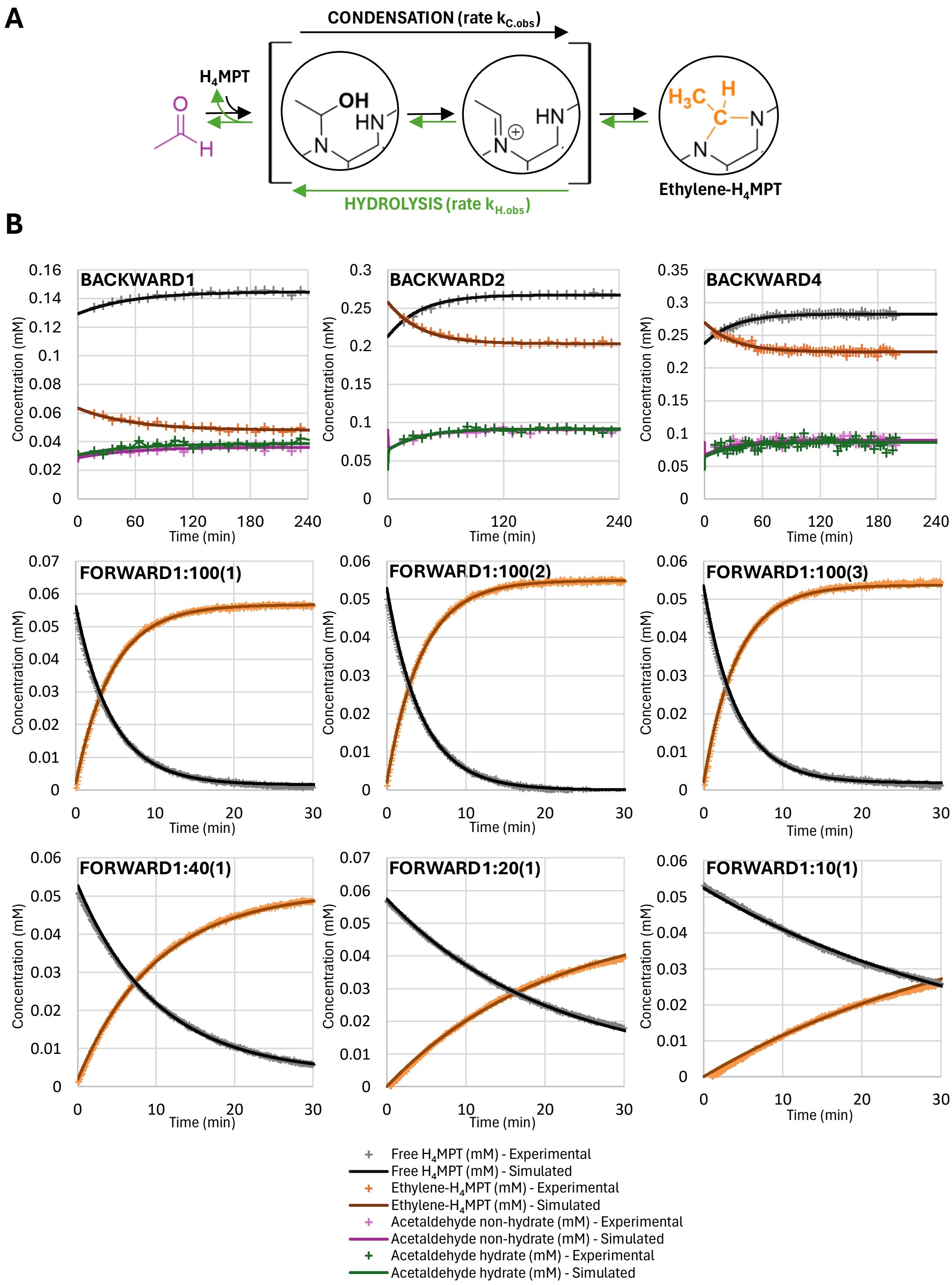

Figure S2: Time courses of kinetic experiment for the condensation of acetaldehyde to H_4MPT and directly following the de-condensation

(**A**) Reaction mechanism. Acetaldehyde is expected to react first with the N^5 position of H_4MPT to a carbinolamine, as proposed for formaldehyde condensation with H_4F[1]. This carbinolamine eliminates water to form the iminium ion that cyclizes to form the ethylene bridge in N^5,N^{10}-

ethylene-H_4MPT. (**B**) Measured kinetics and overlayed simulations of the condensation of acetaldehyde with free H_4MPT, acquired in NMR in the hydrolysis direction (BACKWARD) and in UV in the condensation direction (FORWARD). BACKWARD1: 0.1mM Acetaldehyde with 0.2 mM H_4MPT (molar ratio of 1:2); BACKWARD2 and BACKWARD4: 0.4mM Acetaldehyde with 0.5 mM H_4MPT (molar ratio of 0.8:1); FORWARD1:100(1-3): 0.05 mM H_4MPT with 5 mM acetaldehyde; FORWARD1:40(1): 0.05 mM H_4MPT with 2 mM acetaldehyde; FORWARD1:20(1): 0.05 mM H_4MPT with 1 mM acetaldehyde; FORWARD1:10(1): 0.05 mM H_4MPT with 0.5 mM acetaldehyde. NB: Acetaldehyde concentration refers here to the sum of concentrations of hydrate and non-hydrate species.

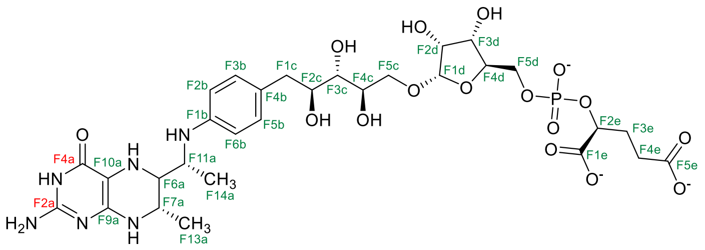

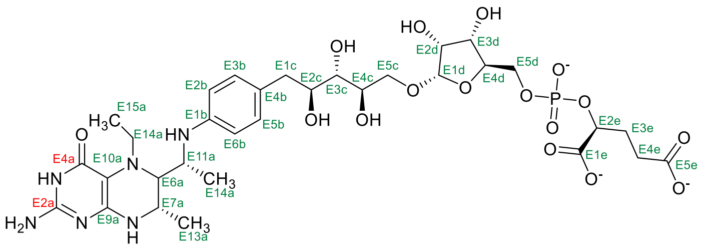

Free H_4MPT

Position	Atom	Shift (ppm)	Range (ppm)	m	J (Hz)
F9a	C	152.170	152.327 .. 152.013	s	/
F10a	C	96.809	96.889 .. 96.708	s	/
F4a	C	/	/	/	/
F7a	C	46.487	46.669 .. 46.308	s	/
	H(1)	3.657	3.697 .. 3.614	m	/
F2a	C	/	/	/	/
F6a	C	58.841	58.979 .. 58.701	s	/
	H(1)	2.922	2.953 .. 2.896	m	/
F13a	C	20.760	20.888 .. 20.633	s	/
	H(3)	1.189	1.201 .. 1.177	d	6.57
F11a	C	52.499	52.679 .. 52.342	s	/
	H(1)	3.421	3.450 .. 3.391	m	/
F12a	C	16.977	17.122 .. 16.833	s	/
	H(3)	1.193	1.205 .. 1.177	d	6.5
F1b	C	143.991	144.172 .. 143.809	s	/
F2b	C	116.671	116.854 .. 116.484	s	/
	H(1)	6.699	6.721 .. 6.673	d	8.53
F6b	C	116.671	116.854 .. 116.484	s	/
	H(1)	6.699	6.721 .. 6.673	d	8.53
F3b	C	130.834	130.703 .. 130.907	s	/
	H(1)	7.068	7.095 .. 7.047	d	8.52
F5b	C	130.834	130.703 .. 130.907	s	/
	H(1)	7.068	7.095 .. 7.047	d	8.52
F4b	C	130.978	131.038 .. 130.913	s	/
F1c	C	37.350	37.486 .. 37.213	s	/
	H'(1)	2.918	2.949 .. 2.893*	dd	13.34, 2.72
	H"(1)	2.518	2.546 .. 2.487	dd	9.78, 14.23
F2c	C	70.752	70.795 .. 70.705[1]	s	/
	H(1)	3.936	3.961 .. 3.901	m	/
F3c	C	74.825	74.863 .. 74.766[1]	s	/
	H(1)	3.646	3.675 .. 3.617	m	/
F4c	C	73.366	73.465 .. 73.270[1]	s	/
	H(1)	3.871	3.904 .. 3.834	m	/
F5c (s)	C	69.544	69.602 .. 69.479	s	/
	H'(1)	3.893	3.920 .. 3.860	m	/
	H"(1)	3.711	3.737 .. 3.684	m	/
F1d	C	102.579	102.621 .. 102.545[1]	s	/
	H(1)	5.076	5.085 .. 5.068	d	6.54
F2d	C	71.656	71.779 .. 71.533	s	/
	H(1)	4.139	4.156 .. 4.120	m	/
F4d	C	84.191	84.278 .. 84.103[1,2]	d	8.51
	H(1)	4.174	4.198 .. 4.156	m	/
F3d	C	70.329	69.084 .. 71.301	s	/
	H(1)	4.082	4.106 .. 4.057	m	/
F5d	C	65.530	65.655 .. 65.403[2]	d	4.79
	H(2)	3.904	3.950 .. 3.867	m	/
F2e	C	76.458	76.601 .. 76.305[2]	d	5.51
	H(1)	4.352	4.378 .. 4.328	ddd	8.83, 6.52, 4.90
F3e	C	30.947	31.084 .. 30.813[2]	d	5
	H(2)	1.932	2.001 .. 1.872	m	/
F4e	C	33.251	33.410 .. 33.093	s	/
	H(2)	2.217	2.291 .. 2.141	m	/
F5e	C	182.528	182.719 .. 182.337	s	/
F1e	C	178.815	179.107 .. 178.524	s	/

***N*^{5}-ethyl-H_4MPT**

Position	Atom	Shift (ppm)	Range (ppm)	m	J (Hz)
E9a	C	153.99	154.096 .. 153.882	s	/
E10a	C	97.023	97.157 .. 96.939	s	/
E4a	C	/	/	/	/
E7a	C	46	46.126 .. 45.874	s	/
	H(1)	3.883	3.925 .. 3.841	m	/
E2a	C	/	/	/	/
E6a	C	59.701	59.826 .. 59.574	s	/
	H(1)	3.336	3.353 .. 3.298	m	/
E13a	C	21.573	21.685 .. 21.462	s	/
	H(3)	1.396	1.418 .. 1.365	d	6.92
E11a	C	56.758	56.959 .. 56.558	s	/
	H(1)	3.085	3.111 .. 3.064	dd	6.42, 10.04
E12a	C	14.84	14.038 .. 16.019	s	/
	H(3)	1.244	1.256 .. 1.234	d	6.46
E1b	C	138.851	139.114 .. 138.593	s	/
E2b	C	120.607	120.764 .. 120.448	s	/
	H(1)	6.878	6.904 .. 6.851	d	8.59
E6b	C	120.607	120.764 .. 120.448	s	/
	H(1)	6.878	6.904 .. 6.851	d	8.59
E3b	C	131.185	131.311 .. 131.082	s	/
	H(1)	7.229	7.255 .. 7.202	d	8.51
E5b	C	131.185	131.311 .. 131.082	s	/
	H(1)	7.229	7.255 .. 7.202	d	8.51
E4b	C	136.585	136.801 .. 136.367	s	/
E1c	C	37.741	37.875 .. 37.607	s	/
	H'(1)	3.004	3.033 .. 2.975	dd	2.89, 14.09
	H"(1)	2.592	2.617 .. 2.559	dd	9.81, 14.21
E2c	C	70.665	70.705 .. 70.626[1]	s	/
	H(1)	3.936	3.961 .. 3.901	m	/
E3c	C	74.934	75.006 .. 74.870[1]	s	/
	H(1)	3.646	3.675 .. 3.617	m	/
E4c	C	73.19	73.274 .. 73.104[1]	s	/
	H(1)	3.871	3.904 .. 3.834	m	/
E5c	C	69.424	69.477 .. 69.362	s	/
	H'(1)	3.893	3.920 .. 3.860	m	/
	H"(1)	3.711	3.737 .. 3.684	m	/
E1d	C	102.519	102.544 .. 102.491	s	/
	H(1)	5.069	5.089 .. 5.048	d	6.61
E2d	C	71.656	71.779 .. 71.533	s	/
	H(1)	4.139	4.156 .. 4.120	m	/
E4d	C	84.235	84.323 .. 84.098[1,2]	d	8.56
	H(1)	4.174	4.198 .. 4.156	m	/
E3d	C	70.228	70.356 .. 70.101	s	/
	H(1)	4.075	4.100 .. 4.056	m	/
E5d	C	65.53	65.655 .. 65.403[2]	d	4.79
	H(2)	3.904	3.950 .. 3.867	m	/
E2e	C	76.458	76.601 .. 76.305[2]	d	5.51
	H(1)	4.352	4.378 .. 4.328	ddd	8.83, 6.52, 4.90
E3e	C	30.947	31.084 .. 30.813[2]	d	5
	H(2)	1.932	2.001 .. 1.872	m	/
E4e	C	33.251	33.410 .. 33.093	s	/
	H(2)	2.217	2.291 .. 2.141	m	/
E5e	C	182.528	182.719 .. 182.337	s	/
E1e	C	178.815	179.107 .. 178.524	s	/
E14a	C	49.695	49.822 .. 49.569	s	/
	H'(1)	3.377	3.414 .. 3.339[3]	m	7.20, 13.20
	H"(1)	3.276	3.306 .. 3.245[3]	m	7.20, 13.20
E15a	C	12.911	13.012 .. 12.810	s	/
	H(3)	1.236	1.269 .. 1.212	t	7.25

Figure S3: Assignment table for ^{1}H- and ^{13}C-NMR signals shifts of H_4MPT and *N*^{5}-ethyl-H_4MPT

Structure of free H_4MPT (left) and *N*^{5}-ethyl-H_4MPT (right) and the corresponding ^{1}H- and ^{13}C-NMR signals shifts deduced from ^{1}H, ^{13}C, HSQC and HMBC experiments (for assignments that are redundant over several spectra and with different shift ranges, only the one with the narrowest range, sharper signals and less overlap is reported) *: Deduced by analogy to E1c'; [1]: Attributed based on peak intensity; [2]: Considered as doublets since in range for PC coupling; [3]: Estimated from spectrum simulation. Quaternary carbon atoms in red could not be unambiguously assigned.

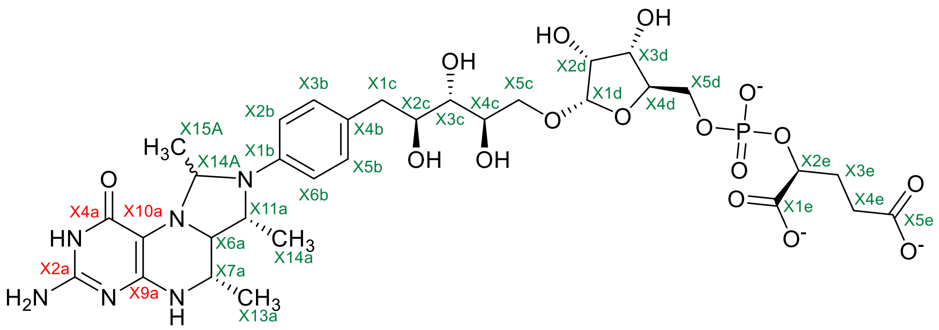

N^5,N^{10}-ethylene-H_4MPT isomer A

Position	Atom	Shift (ppm)	Range (ppm)	m	J(Hz)
A6a	C	66.665	65.874 .. 67.457	/	/
	H(1)	2.624	2.646 .. 2.599	dd	1.63, 9.07
A7a	C	48.778	47.987 .. 49.569	/	/
	H(1)	3.174	3.211 .. 3.135	m	/
A11a	C	54.9	54.110 .. 55.693	/	/
	H(1)	4.008	4.027 .. 3.980	m	/
A12a	C	18.241	17.106 .. 19.450	/	/
	H(3)	1.25	1.256 .. 1.228	d	6.17
A13a	C	17.676	16.519 .. 18.545	/	/
	H(3)	1.266	1.284 .. 1.259	/	/
A14a	C	72.789	71.998 .. 73.580	/	/
	H(1)	5.417	5.456 .. 5.378	q	5.65
A15a	C	17.031	16.005 .. 18.141	/	/
	H(3)	0.892	0.914 .. 0.870	d	5.72
A1b	C	143.429	142.365..144.492	/	/
A2b	C	116.783	115.255 .. 117.955	/	/
	H(2)	6.789	6.809 .. 6.764	d	8.44
A3b	C	130.964	129.541 .. 132.214	/	/
	H(2)	7.19	7.217 .. 7.168	d	8.39
A4b	C	128.51	127.447..129.573	/	/
A5b	C	130.964	129.541 .. 132.214	/	/
	H(2)	7.19	7.217 .. 7.168	d	8.39
A6b	C	116.783	115.255 .. 117.955	/	/
	H(2)	6.789	6.809 .. 6.764	d	8.44
A1c	C	37.014	36.223 .. 37.805	/	/
	H'(1)	2.938	2.983 .. 2.886	m	/
	H''(1)	2.535	2.580 .. 2.480	m	/
A2c	C	70.855	69.688 .. 71.948	/	/
	H(1)	3.943	3.971 .. 3.904	m	/
A3c	C	74.884	74.059 .. 75.709	/	/
	H(1)	3.671	3.713 .. 3.630	m	/
A4c	C	73.434	72.277 .. 74.763	/	/
	H(1)	3.878	3.948 .. 3.834	m	/
A5c	C	69.566	68.741 .. 70.391	/	/
	H'(1)	3.9	3.924 .. 3.858	m	/
	H''(1)	3.719	3.760 .. 3.678	m	/
A1d	C	102.602	101.776 .. 103.427	/	/
	H(1)	5.077	5.100 .. 5.056	m	/
A2d	C	71.661	69.544 .. 73.016	/	/
	H(1)	4.141	4.161 .. 4.121	dt	6.37, 4.15
A3d	C	70.211	69.236 .. 71.722	/	/
	H(1)	4.086	4.104 .. 4.064	m	/
A4d	C	84.231	83.405 .. 85.056	/	/
	H(1)	4.188	4.226 .. 4.168	m	/
A5d	C	65.537	64.367 .. 66.873	/	/
	H(2)	3.921	3.962 .. 3.881	m	/
A2e	C	76.495	75.670 .. 77.321	/	/
	H(1)	4.344	4.377 .. 4.313	m	/
A3e	C	31.051	30.130 .. 32.236	/	/
	H(2)	1.93	1.971 .. 1.889	m	/
A4e	C	33.555	32.750 .. 34.458	/	/
	H(2)	2.206	2.292 .. 2.130	m	/
A5e	C	182.676	179.964..185.403	/	/
A1e	C	178.933	177.765..180.297	/	/

N^5,N^{10}-ethylene-H_4MPT isomer B

Position	Atom	Shift (ppm)	Range (ppm)	m	J(Hz)
B6a	C	66.182	65.391 .. 66.973	/	/
	H(1)	2.958	2.997 .. 2.918	m	/
B7a	C	45.071	44.280 .. 45.863	/	/
	H(1)	3.3	3.340 .. 3.261	/	/
B11a	C	59.253	58.427 .. 60.078	/	/
	H(1)	3.656	3.697 .. 3.615	m	/
B12a	C	19.126	17.777 .. 20.095	/	/
	H(3)	1.17	1.193 .. 1.146	d	6.42
B13a	C	20.254	19.050 .. 21.186	/	/
	H(3)	1.408	1.429 .. 1.399	d	6.31
B14a	C	78.92	77.164 .. 80.957	/	/
	H(1)	4.748	4.803 .. 4.689	m	/
B15a	C	21.091	19.875 .. 21.913	/	/
	H(3)	1.471	1.489 .. 1.462	d	5.84
B1b	C	146.505	145.349..147.608	/	/
B2b	C	113.56	112.734 .. 114.385	/	/
	H(2)	6.673	6.700 .. 6.641	d	8.56
B3b	C	130.964	129.714 .. 132.204	/	/
	H(2)	7.128	7.153 .. 7.106	d	8.57
B4b	C	128.51	127.447..129.573	/	/
B5b	C	130.964	129.714 .. 132.204	/	/
	H(2)	7.128	7.153 .. 7.106	d	8.57
B6b	C	113.56	112.734 .. 114.385	/	/
	H(2)	6.673	6.700 .. 6.641	d	8.56
B1c	C	37.014	36.223 .. 37.805	/	/
	H'(1)	2.938	2.983 .. 2.886	m	/
	H''(1)	2.535	2.580 .. 2.480	m	/
B2c	C	70.855	69.688 .. 71.948	/	/
	H(1)	3.943	3.971 .. 3.904	m	/
B3c	C	74.884	74.059 .. 75.709	/	/
	H(1)	3.671	3.713 .. 3.630	m	/
B4c	C	73.434	72.277 .. 74.763	/	/
	H(1)	3.878	3.948 .. 3.834	m	/
B5c	C	69.566	68.741 .. 70.391	/	/
	H'(1)	3.9	3.924 .. 3.858	m	/
	H''(1)	3.719	3.760 .. 3.678	m	/
B1d	C	102.602	101.776 .. 103.427	/	/
	H(1)	5.077	5.100 .. 5.056	m	/
B2d	C	71.661	69.544 .. 73.016	/	/
	H(1)	4.141	4.161 .. 4.121	dt	6.37, 4.15
B3d	C	70.211	69.236 .. 71.722	/	/
	H(1)	4.086	4.104 .. 4.064	m	/
B4d	C	84.231	83.405 .. 85.056	/	/
	H(1)	4.188	4.226 .. 4.168	m	/
B5d	C	65.537	64.367 .. 66.873	/	/
	H(2)	3.921	3.962 .. 3.881	m	/
B2e	C	76.495	75.670 .. 77.321	/	/
	H(1)	4.344	4.377 .. 4.313	m	/
B3e	C	31.051	30.130 .. 32.236	/	/
	H(2)	1.93	1.971 .. 1.889	m	/
B4e	C	33.555	32.750 .. 34.458	/	/
	H(2)	2.206	2.292 .. 2.130	m	/
B5e	C	182.676	179.964..185.403	/	/
B1e	C	178.933	177.765..180.297	/	/

Figure S4: Assignment table for ^{1}H- and ^{13}C-NMR signals shifts of of N^5,N^{10}-ethylene-H_4MPT diastereoisomers

Structure of N^5,N^{10}-ethylene-H_4MPT and the corresponding ^{1}H- and ^{13}C-NMR signals shifts attributed to the isomer A (left) and B (right) deduced from ^{1}H, ^{13}C, HSQC and HMBC experiments (for assignments that are redundant over several spectra and with different shift ranges, only the narrowest range is reported). Quaternary carbon atoms in red could not be unambiguously assigned.

Derivative	λ_{max} (nm)	Absorbance (AU)
H_4MPT	303	0.419
Methylene-H_4MPT	287	0.754
Methyl-H_4MPT	294	0.820
Ethylene-H_4MPT	287	0.727
Ethyl-H_4MPT	290	0.811

Figure S5: UV absorbance values at λ_{max} for H4MPT derivatives

List of UV absorbance values of synthesized H_4MPT derivatives at their respective local extrema in the range 270-310 nm (λ_{max}). See figure Fig.3B for complete spectra.

Compound	Experiment	H_2	H_2+Acetaldehyde	N_2	N_2+Acetaldehyde	No Treatment
H_4MPT	H_2	/				
H_4MPT	H_2+Acetaldehyde	0.984996	/			
H_4MPT	N_2	0.000001	0.000000	/		
H_4MPT	N_2+Acetaldehyde	0.017367	0.000012	0.465624	/	
H_4MPT	No Treatment	0.000001	0.000000	1.000000	0.602720	/
Ethyl-H_4MPT	H_2	/				
Ethyl-H_4MPT	H_2+Acetaldehyde	0.442811	/			
Ethyl-H_4MPT	N_2	0.000000	0.000000	/		
Ethyl-H_4MPT	N_2+Acetaldehyde	0.000005	0.000000	0.000650	/	
Ethyl-H_4MPT	No Treatment	0.000000	0.000000	1.000000	0.000021	/
Ethylene-H_4MPT	H_2	/				
Ethylene-H_4MPT	H_2+Acetaldehyde	0.999999	/			
Ethylene-H_4MPT	N_2	0.012949	0.000235	/		
Ethylene-H_4MPT	N_2+Acetaldehyde	0.908176	0.201424	0.869779	/	
Ethylene-H_4MPT	No Treatment	0.000290	0.000003	1.000000	0.185284	/

Experiment	Ethylene-H_4MPT vs Ethyl-H_4MPT	H_4MPT vs Ethyl-H_4MPT	H_4MPT vs Ethylene-H_4MPT
H_2	0.000	0.000	1.000
H_2+Acetaldehyde	0.000	0.000	1.000
N_2	0.001	0.000	0.142
N_2+Acetaldehyde	0.760	1.000	0.471
No Treatment	0.000	0.000	0.736

Compound	Experiment	H_4MPT Standard	H_4MPT+Acetaldehyde+H_2
H_4MPT	H_4MPT Standard	/	0.000
H_4MPT	H_4MPT+Acetaldehyde+H_2	0.000	/
Ethyl-H_4MPT	H_4MPT Standard	/	1.000
Ethyl-H_4MPT	H_4MPT+Acetaldehyde+H_2	1.000	/
Ethylene-H_4MPT	H_4MPT Standard	/	0.000
Ethylene-H_4MPT	H_4MPT+Acetaldehyde+H_2	0.000	/

Experiment	Ethylene-H_4MPT vs Ethyl-H_4MPT	H_4MPT vs Ethyl-H_4MPT	H_4MPT vs Ethylene-H_4MPT
H_4MPT Standard	1.000	0.000	0.000
H_4MPT+Acetaldehyde+H_2	0.000	1.000	0.000

Figure S6: Statistical analysis of *in vitro* cell lysate experiments

Numerical data and statistical evaluation (p-values) for the *in vitro* cell-lysate experiments described in Figure 5 (top) and in Figure S8 (bottom).

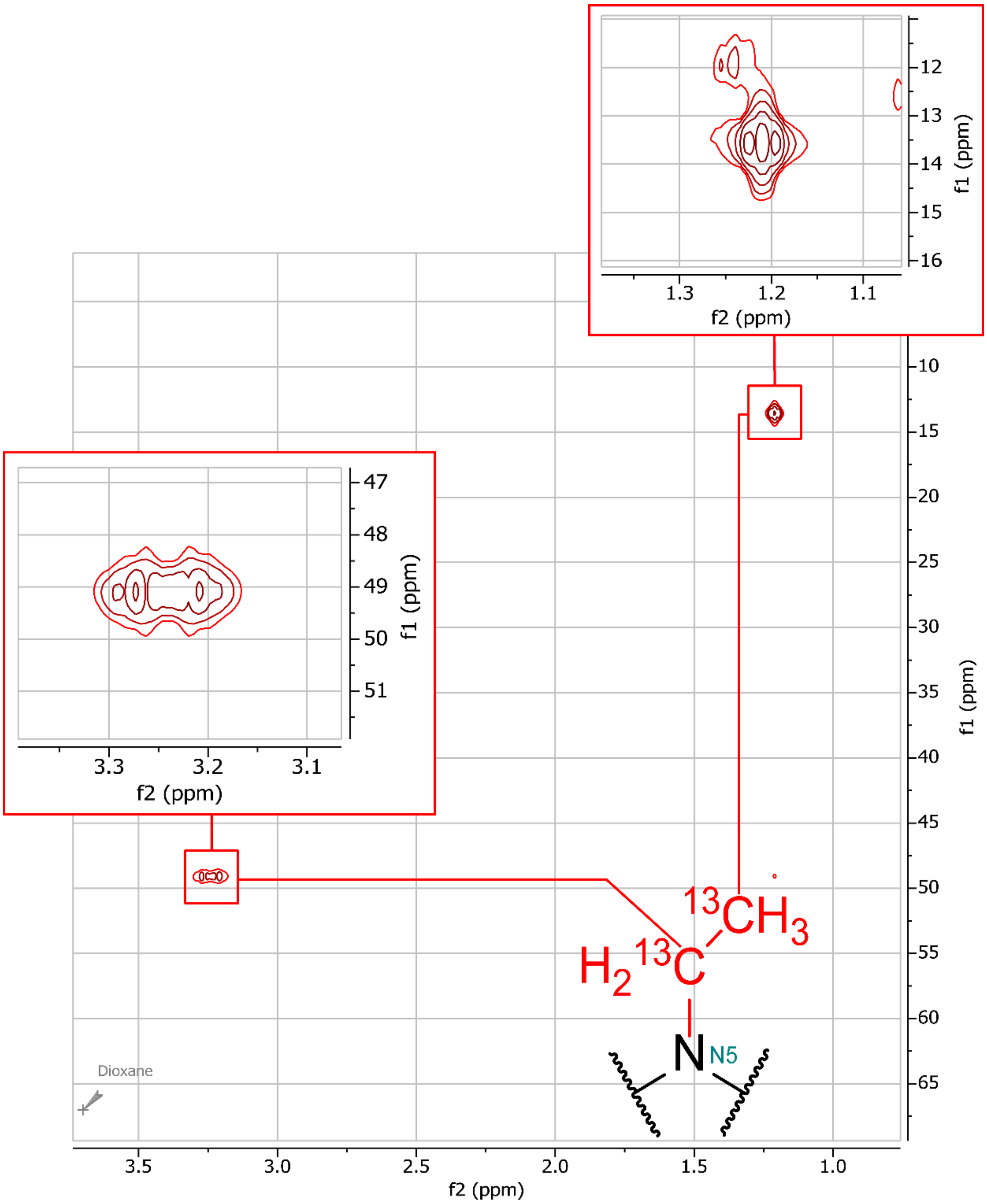

Figure S7: HSQC-spectrum of *N*^{5}-$^{13}C_2$-ethyl-H_4MPT from cell lysates assays with $^{13}C_2$-acetaldehyde

Detection of *N*^{5}-$^{13}C_2$-ethyl-H_4MPT in cell lysates complemented with $^{13}C_2$-acetaldehyde and H_2 in HSQC-NMR. The *N*^{5}-$^{13}C_2$-ethyl group leads to intense signals matching the ethyl group signals in unlabeled *N*^{5}-ethyl-H_4MPT (see section I-1), whereby the unlabeled carbons are not showing up in the magnification applied here.

1 – Characterization data for H_4MPT and *N*^{5}-ethyl-H_4MPT in a ca. 1:1 mixture

This sub-section collects the individual NMR and MS spectra that allow the identification of free H_4MPT and *N*^{5}-ethyl-H_4MPT. For NMR data, the signals are summarized in figure S3. For chemical shifts that can be obtained from different NMR experiments (e.g. a ^{13}C NMR shift can be deduced either from HSQC or from ^{13}C), the value chosen for the complication is the one that is more clearly defined (sharper signals/less overlap); e.g. typically ^{13}C instead of HSQC.

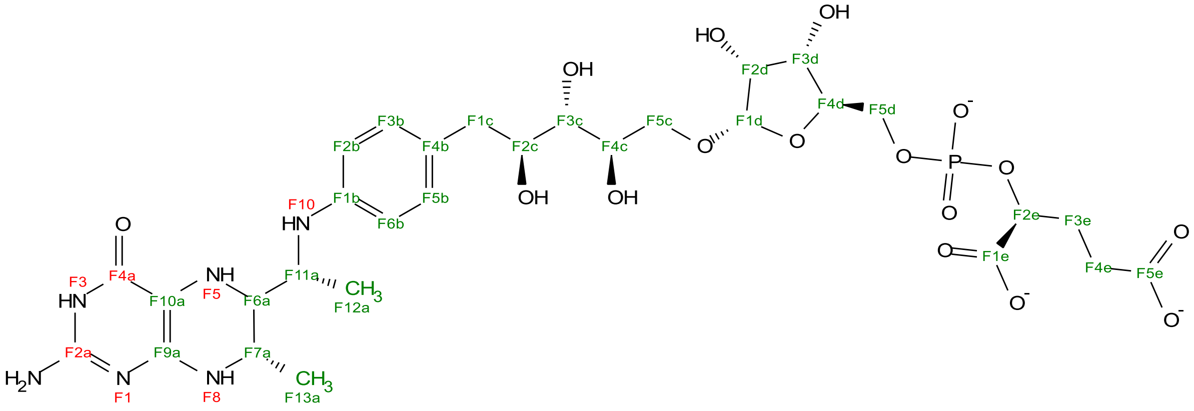

Figure S8 : Structure and atom labelling of H_4MPT used for spectra analysis.

^{13}C or ^{1}H signals that could be assigned are in green, positions for which there are no signals or where the signals could not be unambiguously assigned are labelled in red.

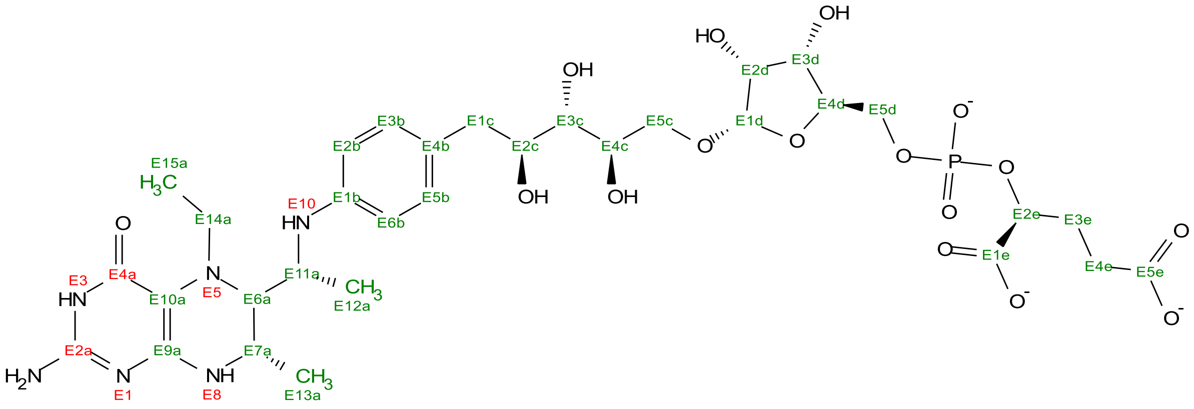

Figure S9: Structure and atom labelling of *N*^{5}-ethyl- H_4MPT used for spectra analysis.

^{13}C or ^{1}H signals that could be assigned are in green, positions for which there are no signals or where the signals could not be unambiguously assigned are labelled in red.

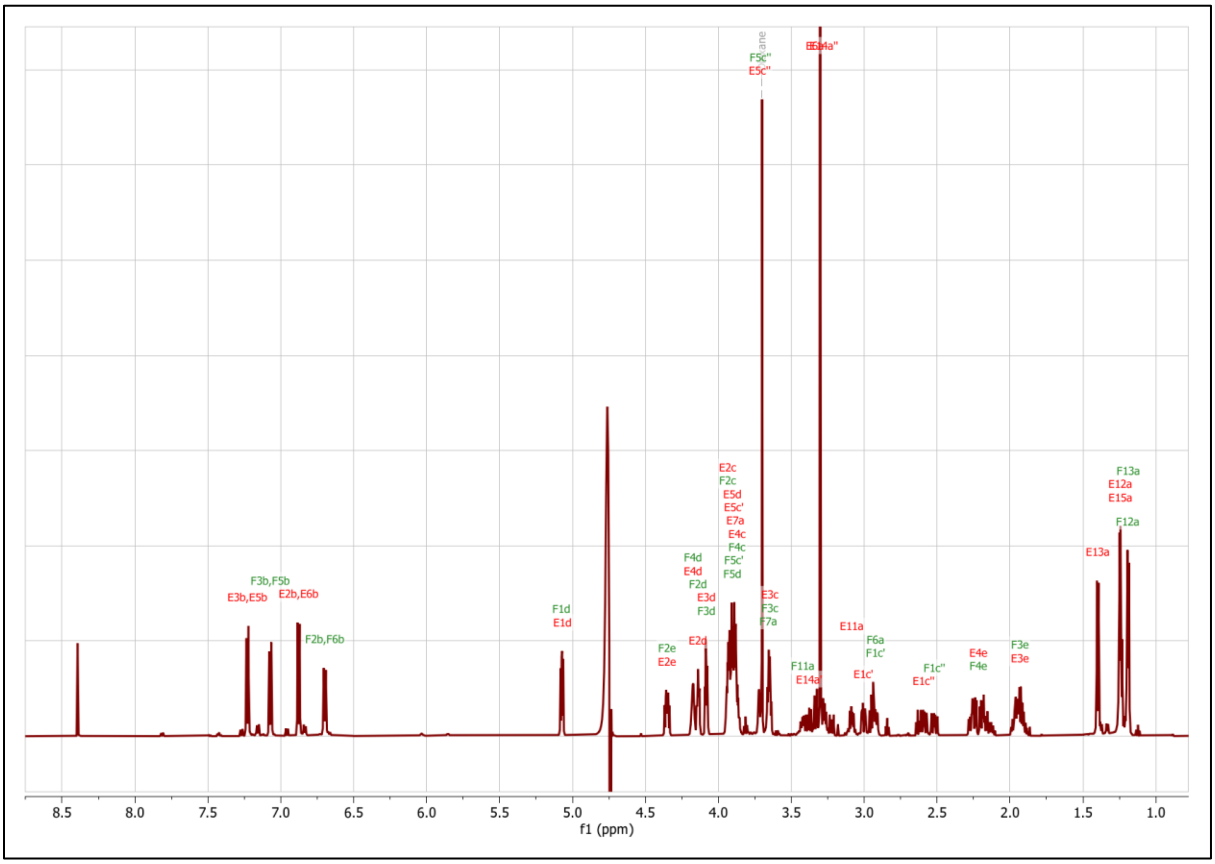

n°	Name	Shift	Range	H's	J's
1	Formate (s)	8.393	8.415 .. 8.371	1	/
2	E3b,E5b (d)	7.229	7.255 .. 7.202	2	8.51
3	F3b,F5b (d)	7.072	7.099 .. 7.045	2	8.52
4	E2b,E6b (d)	6.878	6.904 .. 6.851	2	8.59
5	F2b,F6b (d)	6.698	6.717 .. 6.681	2	8.53
6	F1d (d)	5.076	5.085 .. 5.068	1	6.54
7	E1d (d)	5.069	5.089 .. 5.048	1	6.61
8	E2e,F2e (ddd)	4.352	4.378 .. 4.328	2	8.83, 6.52, 4.90
9	E4d,F4d (m)	4.174	4.198 .. 4.156	2	/
10	E2d,F2d (m)	4.139	4.156 .. 4.120	2	/
11	E3d,F3d (m)	4.082	4.106 .. 4.057	2	/
12	E2c,F2c,E5d,F5d,E5c',F5c',E4c,F4c,E7a (m)	3.905	3.961 .. 3.844	11	/
13	E5c'',F5c'' (m)	3.722	3.740 .. 3.691	2	/
14	Dioxane (s)	3.700	3.715 .. 3.685	8	/
15	E3c,F3c,F7a (m)	3.650	3.679 .. 3.621	3	/
16	F11a (m)	3.418	3.444 .. 3.388	1	/
17	E14a' (m)	3.375	3.411 .. 3.335	1	/
18	E6a (m)	3.332	3.367 .. 3.308	1	/
19	Methanol (s)	3.301	3.310 .. 3.292	3	/
20	E14a'' (m)	3.277	3.307 .. 3.248	1	/
21	E11a (dd)	3.085	3.111 .. 3.064	1	6.42, 10.04
22	E1c' (dd)	3.004	3.033 .. 2.975	1	2.89, 14.09
23	F6a (m)	2.939	2.971 .. 2.889	1	/
24	F1c' (dd)	2.921	2.953 .. 2.892	1	13.34, 2.72*
25	E1c'' (dd)	2.592	2.617 .. 2.559	1	9.81, 14.21
26	F1c'' (dd)	2.518	2.546 .. 2.487	1	9.78, 14.23
27	E4e,F4e (m)	2.200	2.304 .. 2.093	4	/
28	E3e,F3e (m)	1.930	2.016 .. 1.872	4	/
29	Acetate (s)	1.864	1.872 .. 1.857	3	/
30	E13a (d)	1.398	1.434 .. 1.366	3	6.92
31	E12a (d)	1.244	1.256 .. 1.234	3	6.46
32	E15a (t)	1.243	1.270 .. 1.216	3	7.25
33	F12a (d)	1.193	1.205 .. 1.177	3	/
34	F13a (d)	1.189	1.201 .. 1.177	3	6.57

Figure S10: 600 MHz ^{1}H-NMR spectrum with assignments

^{1}H-NMR spectrum (top) and corresponding assignments (bottom) of a mixture of free H_4MPT (assignments in green) and *N*^{5}-ethyl-H_4MPT (assignments in red). *: Deduced by analogy to E1c'

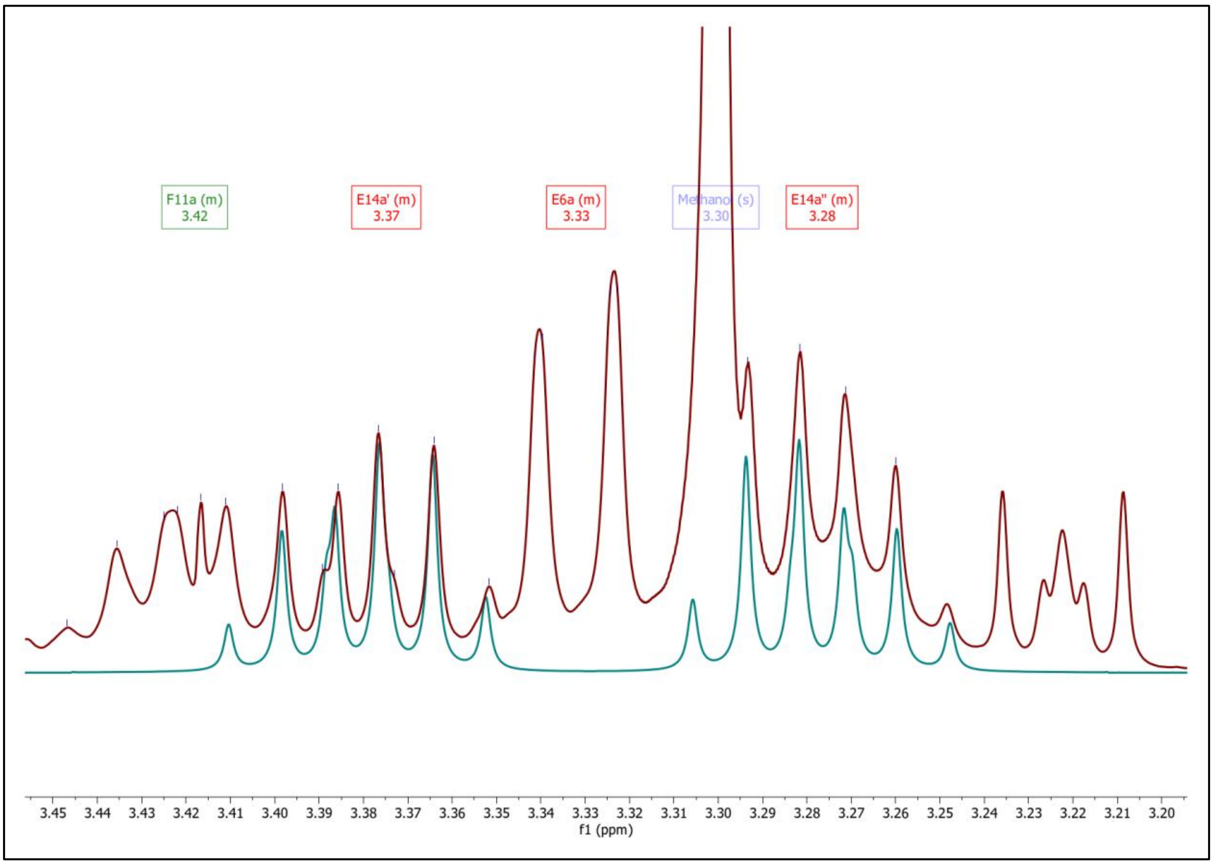

Figure S11: ^{1}H-NMR Simulation of the ethyl group of *N*^{5}-ethyl-H_4MPT and overlap with ^{1}H-NMR spectrum

^{1}H-NMR spectrum overlay of a mixture of free H_4MPT and *N*^{5}-ethyl-H_4MPT (dark red) and the spectrum simulation of *N*^{5}-ethyl-H_4MPT signals 14A' and 14A'' (turquoise).

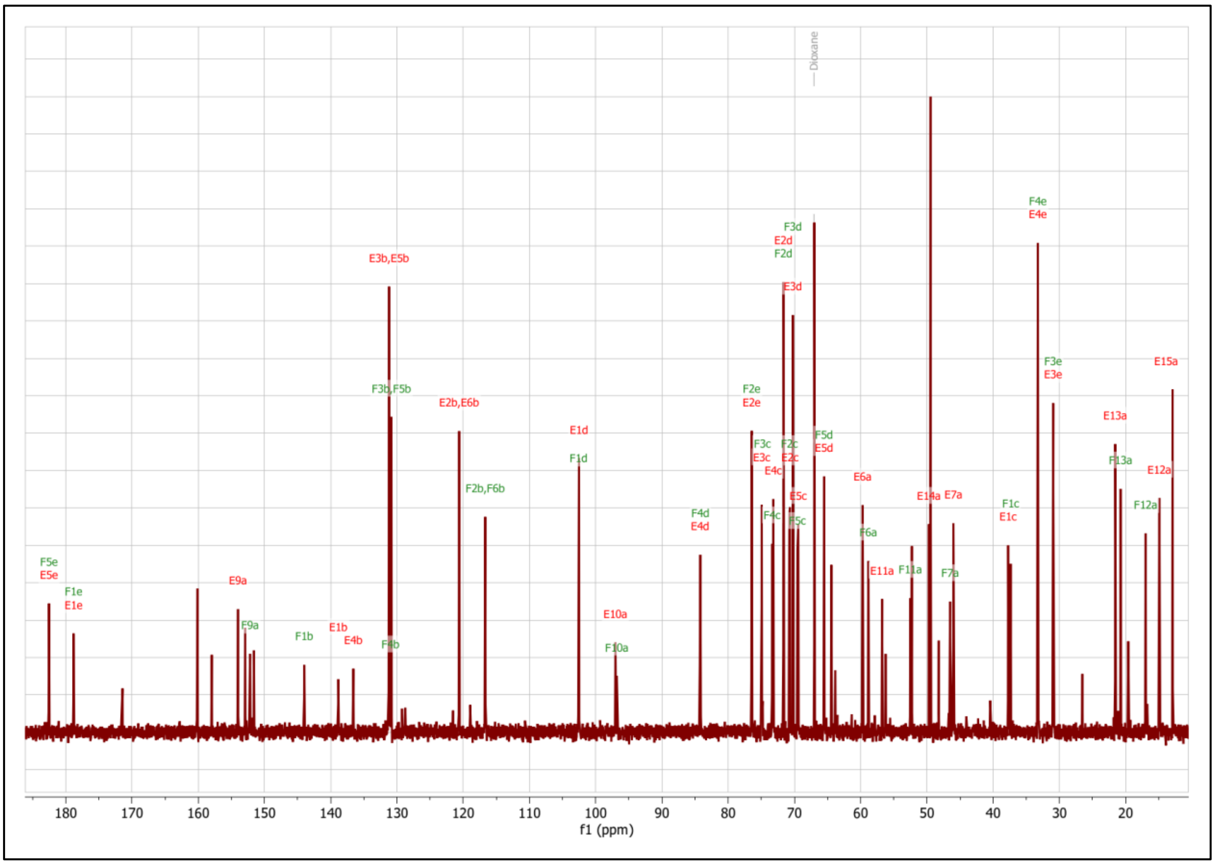

n°	Name	Shift	Range	C's	J's
1	E5e,F5e (s)	182.528	182.719 .. 182.337	2	/
2	E1e,F1e (s)	178.815	179.107 .. 178.524	2	/
3	E9a (s)	153.990	154.096 .. 153.882	1	/
4	F9a (s)	152.170	152.327 .. 152.013	1	/
5	F1b (s)	143.991	144.172 .. 143.809	1	/
6	E1b (s)	138.851	139.114 .. 138.593	1	/
7	E4b (s)	136.585	136.801 .. 136.367	1	/
8	E3b,E5b (s)	131.185	131.311 .. 131.082	2	/
9	F4b (s)	130.978	131.038 .. 130.913	1	/
10	F3b,F5b (s)	130.834	130.907 .. 130.703	2	/
11	E2b,E6b (s)	120.607	120.764 .. 120.448	2	/
12	F2b,F6b (s)	116.671	116.854 .. 116.484	2	/
13	F1d (s)	102.579[1]	102.621 .. 102.545	1	/
14	E1d (s)	102.519[1]	102.544 .. 102.491	1	/
15	E10a (s)	97.023	97.157 .. 96.939	1	/
16	F10a (s)	96.809	96.889 .. 96.708	1	/
17	E4d (d)	84.235[1,2]	84.323 .. 84.098	1	8.56
18	F4d (d)	84.191[1,2]	84.278 .. 84.103	1	8.51
19	E2e,F2e (d)	76.458[1,2]	76.601 .. 76.305	2	5.51
20	E3c (s)	74.934[1]	75.006 .. 74.870	1	/
21	F3c (s)	74.825[1]	74.863 .. 74.766	1	/
22	F4c (s)	73.366[1]	73.465 .. 73.270	1	/
23	E4c (s)	73.190[1]	73.274 .. 73.104	1	/
24	E2d,F2d (s)	71.656	71.779 .. 71.533	2	/

n°	Name	Shift	Range	C's	J's
25	F2c (s)	70.752	70.795 .. 70.705	1	/
26	E2c (s)	70.665	70.705 .. 70.626	1	/
27	E3d,F3d (s)	70.228	70.356 .. 70.101	2	/
28	F5c (s)	69.544	69.602 .. 69.479	1	/
29	E5c (s)	69.424	69.477 .. 69.362	1	/
30	Dioxane (s)	67.000	67.087 .. 66.913	1	/
31	E5d,F5d (d)	65.53[2]	65.655 .. 65.403	1	4.79
32	E6a (s)	59.701	59.826 .. 59.574	1	/
33	F6a (s)	58.841	58.979 .. 58.701	1	/
34	E11a (s)	56.758	56.959 .. 56.558	1	/
35	F11a (s)	52.499	52.679 .. 52.342	1	/
36	E14a (s)	49.695	49.822 .. 49.569	1	/
37	Methanol (s)	49.445	49.531 .. 49.358	1	/
38	F7a (s)	46.487	46.669 .. 46.308	1	/
39	E7a (s)	46.000	46.126 .. 45.874	1	/
40	E1c (s)	37.741	37.875 .. 37.607	1	/
41	F1c (s)	37.350	37.486 .. 37.213	1	/
42	E4e,F4e (s)	33.251	33.410 .. 33.093	2	/
43	E3e,F3e (d)	30.947[2]	31.084 .. 30.813	2	5.00
44	E13a (s)	21.573	21.685 .. 21.462	1	/
45	F13a (s)	20.760	20.888 .. 20.633	1	/
46	F12a (s)	16.977	17.122 .. 16.833	1	/
47	E12a (s)	14.908	15.046 .. 14.769	1	/
48	E15a (s)	12.911	13.012 .. 12.810	1	/

Figure S12: 150 MHz ^{13}C-NMR spectrum with assignments

^{13}C NMR spectrum (top) and corresponding assignments (bottom) of a mixture of free H_4MPT (assignments in green) and N^5-ethyl-H_4MPT (assignments in red). [1]: Attributed based on peak intensity; [2]: Considered as doublets since in range for PC coupling.

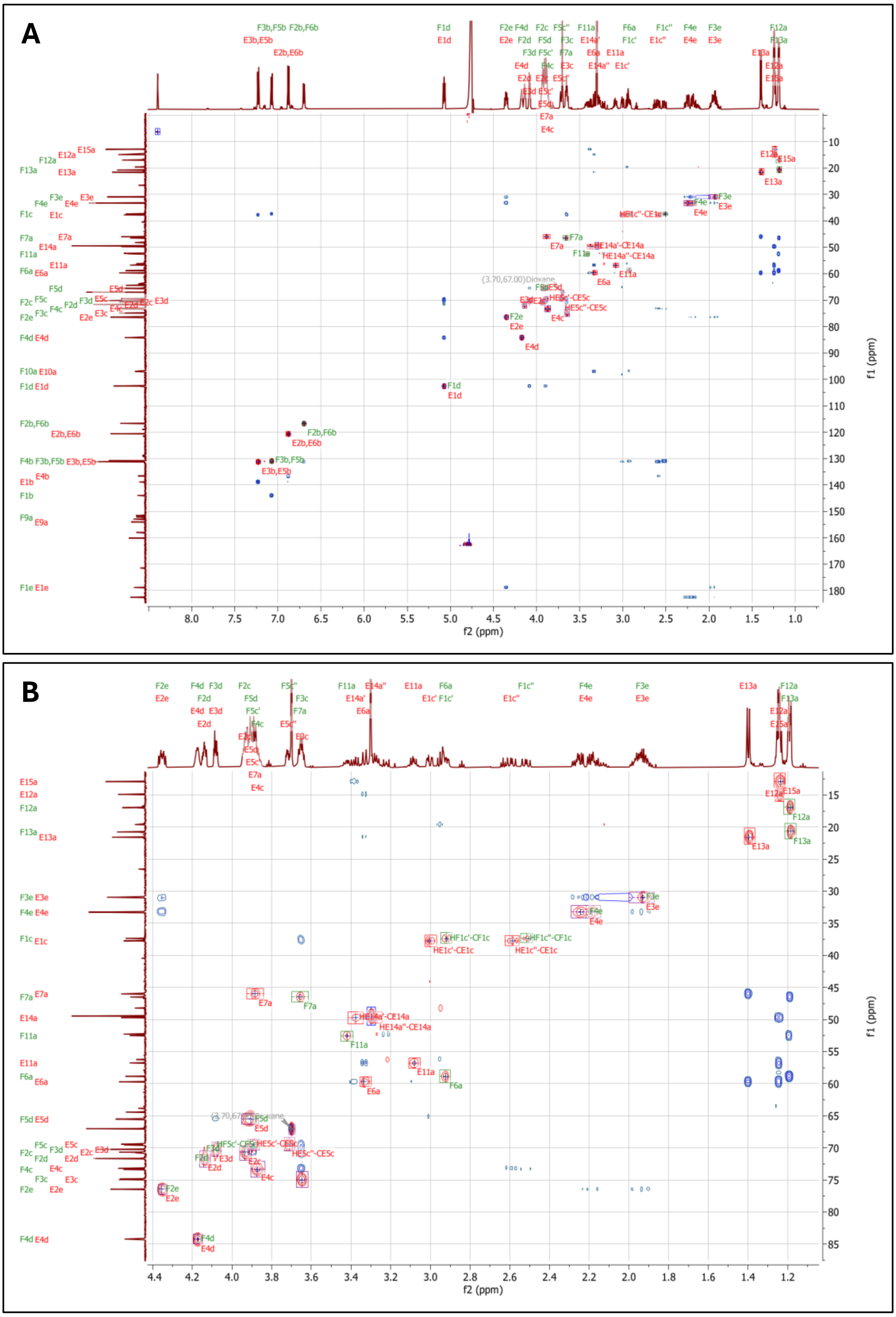

Figure S13: HSQC and HMBC spectra overlays

Overlap of HSQC (red dots) and HMBC (blue dots) NMR spectra of a mixture of free H_4MPT (assignments in green) and *N*^{5}-ethyl-H_4MPT (assignments in red). f1 and f2 traces correspond to the ^{1}H and ^{13}C NMR spectra shown in Figure S10 and Figure S12). A: Whole spectrum; B: Expansion of the region between 10 and 90 ppm for ^{13}C and between 1.1 and 4.4 for ^{1}H.

n°	Name	C Shift	C Range	H Shift	H Range
1	Formate	6.291	5.023 .. 7.460	8.394	8.420 .. 8.370
2	Dioxane	67.103	66.301 .. 67.905	3.699	3.715 .. 3.681
3	Methanol	49.594	48.073 .. 50.875	3.300	3.319 .. 3.277
4	E4d,F4d	84.202	83.400 .. 85.004	4.172	4.195 .. 4.147
5	E2d,F2d	71.620	70.444 .. 73.016	4.139	4.162 .. 4.110
6	E2e,F2e	76.459	75.657 .. 77.261	4.354	4.371 .. 4.324
7	E5d,F5d	65.490	64.688 .. 66.292	3.904	3.950 .. 3.867
8	E2c,F2c	70.652	69.697 .. 71.935	3.936	3.961 .. 3.901
9	E4c,F4c	73.394	72.116 .. 74.534	3.871	3.904 .. 3.834
10	E3c,F3c	75.007	73.656 .. 76.170	3.646	3.675 .. 3.617
11	HE5c''-CE5c,HF5c''-CF5c	69.523	68.384 .. 70.445	3.711	3.737 .. 3.684
12	E6a	59.683	58.881 .. 60.485	3.336	3.353 .. 3.298
13	E11a	56.780	55.978 .. 57.582	3.081	3.110 .. 3.050
14	F11a	52.586	51.784 .. 53.388	3.421	3.450 .. 3.391
15	E7a	45.972	45.170 .. 46.774	3.883	3.925 .. 3.841
16	F7a	46.456	45.654 .. 47.258	3.657	3.697 .. 3.614
17	E4e,F4e	33.229	32.233 .. 34.182	2.217	2.291 .. 2.141
18	E3e,F3e	30.971	30.169 .. 31.773	1.932	2.001 .. 1.872
19	HE5c'-CE5c,HF5c'-CF5c	69.523	68.647 .. 70.290	3.893	3.920 .. 3.860
20	HE1c'-CE1c	37.746	36.852 .. 38.631	3.005	3.026 .. 2.969
21	HF1c'-CF1c	37.423	36.429 .. 38.128	2.918	2.949 .. 2.893
22	HE1c''-CE1c	37.746	36.881 .. 38.471	2.585	2.625 .. 2.553
23	HF1c''-CF1c	37.355	36.582 .. 38.019	2.515	2.545 .. 2.485
24	HE14a''-CE14a	49.590	48.793 .. 50.529	3.276	3.306 .. 3.245
25	E1d,F1d	102.591	101.682 .. 103.470	5.072	5.093 .. 5.049
26	E3d,F3d	70.329	69.084 .. 71.301	4.075	4.100 .. 4.056
27	E3b,E5b	131.303	130.470 .. 132.105	7.226	7.253 .. 7.200
28	F3b,F5b	130.981	130.179 .. 131.783	7.068	7.095 .. 7.047
29	E2b,E6b	120.657	119.855 .. 121.459	6.872	6.904 .. 6.851
30	F2b,F6b	116.686	115.984 .. 117.588	6.699	6.721 .. 6.673
31	HE14a'-CE14a	49.682	48.833 .. 50.651	3.377	3.414 .. 3.339
32	F13a	20.647	19.386 .. 21.833	1.184	1.213 .. 1.158
33	F12a	16.937	15.644 .. 18.013	1.187	1.220 .. 1.163
34	E12a	14.840	14.038 .. 16.019	1.239	1.263 .. 1.220
35	E15a	12.904	11.630 .. 13.753	1.236	1.269 .. 1.212
36	E13a	21.615	20.137 .. 22.635	1.396	1.418 .. 1.365
37	F6a	58.877	57.844 .. 59.846	2.922	2.953 .. 2.900

Figure S14: Assignment table for HSQC spectrum

Assignments table of free H_4MPT (assignments in green) and N^5-ethyl-H_4MPT (assignments in red) signals in the HSQC NMR spectrum (see Figure S13).

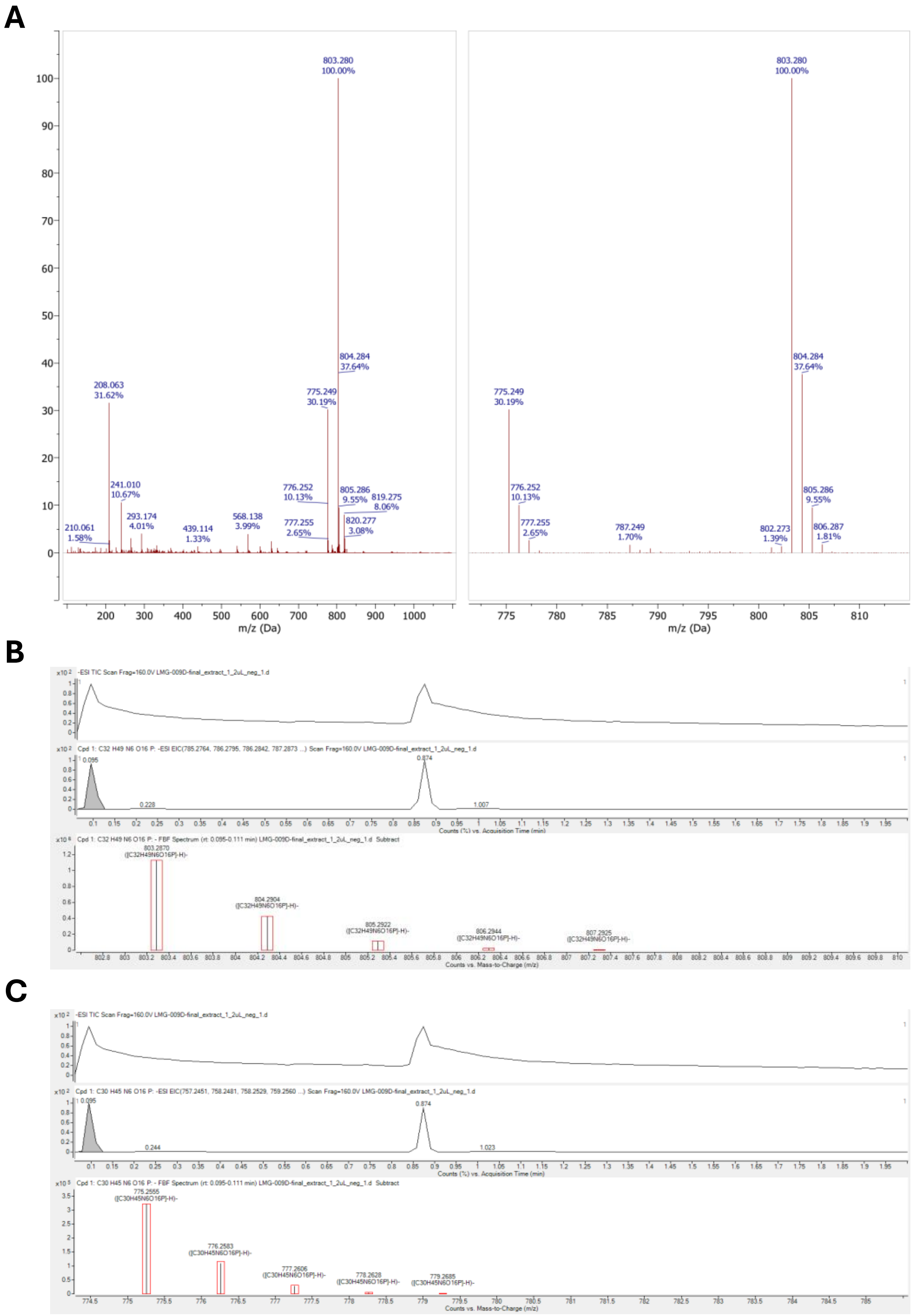

Figure S15: MS-QTOF spectrum

MS centroid spectra of a mixture of free H_4MPT and *N*[5]-ethyl-H_4MPT (MS-QTOF, ESI negative). (**A**) Left: Global spectrum; Right: Zoom on peaks at 775.249 and 803.280 m/z corresponding to Free H_4MPT and *N*[5]-ethyl-H_4MPT respectively. (**B**) Isotope peaks detected for *N*[5]-ethyl-H_4MPT. Main mass calculated for [C32H49N6O16P - H]-: 803.2870, found: 803.2870 (Δ = 0.07 ppm) (**C**) Isotope peaks detected for free H_4MPT. Main mass calculated for [C30H45N6O16P - H]-: 775.2557, found: 775.2555 (Δ = -0.33 ppm).

2 – Characterization data for *N*^{5},*N*^{10}-ethylene-H_4MPT diastereoisomers

This sub-section collects the individual NMR and MS spectra that allow the identification of *N*^{5},*N*^{10}-ethylene-H_4MPT diastereoisomers. For NMR data, the signals are summarized in Figure S4. For chemical shifts that can be obtained from different NMR experiments (e.g. a ^{13}C NMR shift can be deduced either from HSQC or from ^{13}C), the value chosen for the complication is the one that is more clearly defined (sharper signals/less overlap); e.g. typically ^{13}C instead of HSQC.

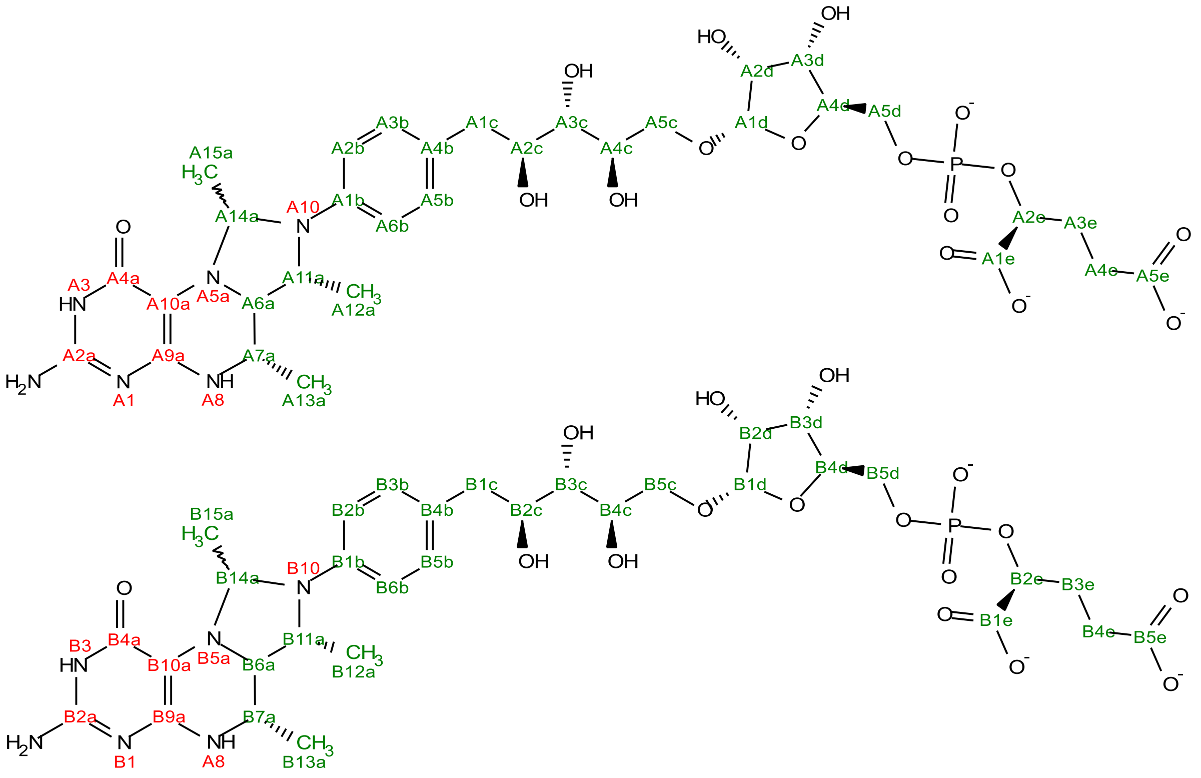

Figure S16: Structure and atom labelling of *N*^{5},*N*^{10}-ethylene-H_4MPT (isomers A and B) used for spectra analysis.

The absolute configuration at carbon 14a to isomers A or B could not be attributed. ^{13}C or ^{1}H signals that could be assigned are in green, positions for which there are no signals, or where the signals could not be unambiguously assigned are labelled in red.

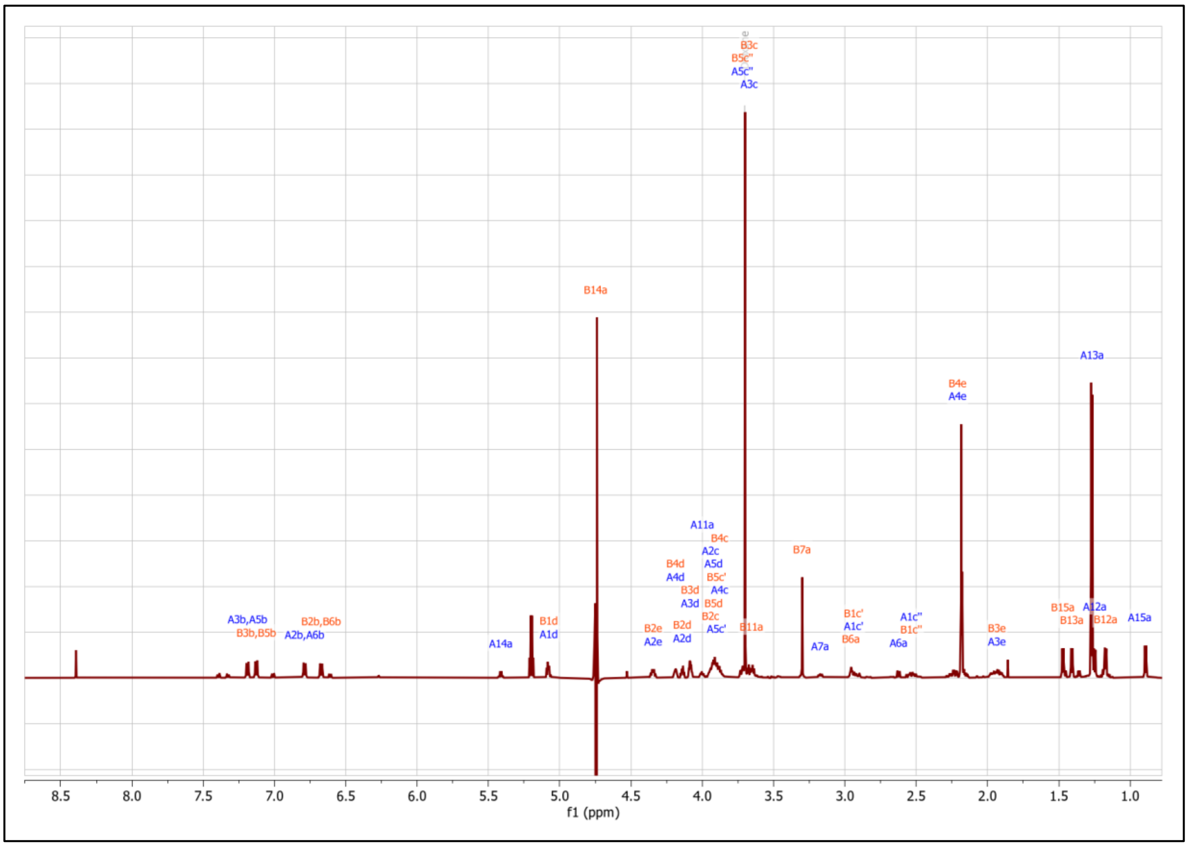

n°	Name	Shift	Range	H's	Class	J's
1	Acetaldehyde non-hydrate	9.62	9.630 .. 9.599	1	q	3.00
2	14a (methenyl-H_4MPT)	8.99	8.997 .. 8.978	1	s	/
3	Formate	8.39	8.403 .. 8.383	1	s	/
4	3b, 5b (methenyl-H_4MPT)	7.39	7.412 .. 7.378	2	d	8.51
5	2b, 6b (methenyl-H_4MPT)	7.33	7.339 .. 7.315	2	d	8.56
6	A3b,A5b	7.19	7.217 .. 7.168	2	d	8.39
7	B3b,B5b	7.13	7.153 .. 7.106	2	d	8.57
8	3b,5b (free-H_4MPT)	7.01	7.028 .. 6.992	2	d	8.22
9	A2b,A6b	6.79	6.809 .. 6.764	2	d	8.44
10	B2b,B6b	6.67	6.700 .. 6.641	2	d	8.56
11	2b,6b (free-H_4MPT)	6.61	6.634 .. 6.584	2	d	8.38
12	A14a	5.41	5.460 .. 5.370	1	q	5.65, 5.65, 5.68
13	Acetaldehyde hydrate	5.2	5.220 .. 5.176	1	q	5.19, 5.19, 5.20
14	A1d,B1d	5.08	5.100 .. 5.056	2	m	/
15	A2e,B2e	4.34	4.377 .. 4.313	2	m	/
16	A4d,B4d	4.19	4.226 .. 4.168	2	m	/
17	A2d,B2d	4.14	4.161 .. 4.121	2	dt	6.37, 4.15
18	A3d,B3d	4.09	4.104 .. 4.064	2	m	/
19	A11a	4.01	4.027 .. 3.980	1	m	/
20	A2c,B2c,A5d,B5d,A5c',B5c',A4c,B4c	3.91	3.967 .. 3.834	10	m	/
21	Dioxane	3.7	3.710 .. 3.687	8	s	/
22	A3c,B3c,A5c",B5c",B11a	3.68	3.749 .. 3.610	5	m	/
23	Methanol	3.3	3.320 .. 3.281	3	s	/
24	A7a	3.17	3.211 .. 3.135	1	m	/
25	A1c",B1c",B6a	2.93	2.990 .. 2.864	3	m	/
26	A6a	2.62	2.646 .. 2.599	1	dd	1.63, 9.07
27	A1c",B1c"	2.53	2.578 .. 2.495	2	m	15.36, 25.08
28	A4e,B4e	2.21	2.298 .. 2.117	4	m	/
29	Acetaldehyde hydrate	2.18	2.188 .. 2.177	3	d	2.97
30	A3e,B3e	1.93	2.006 .. 1.870	4	m	/
31	Acetate	1.86	1.866 .. 1.847	3	s	/
32	B15a	1.47	1.489 .. 1.462	3	d	5.84
33	B13a	1.41	1.429 .. 1.399	3	d	6.31
34	A13a, Acetaldehyde non-hydrate	1.27	1.282 .. 1.257	6	d	5.21
35	A12a	1.25	1.258 .. 1.229	3	d	6.17
36	B12a	1.17	1.196 .. 1.148	3	d	6.42
37	A15a	0.89	0.928 .. 0.873	3	d	5.72

Figure S17: 600 MHz ^{1}H-NMR spectrum

^{1}H-NMR spectrum (top) and corresponding assignments (bottom) of *N*^{5},*N*^{10}-ethylene-H_4MPT diastereoisomers A (blue labels) and B (orange labels).

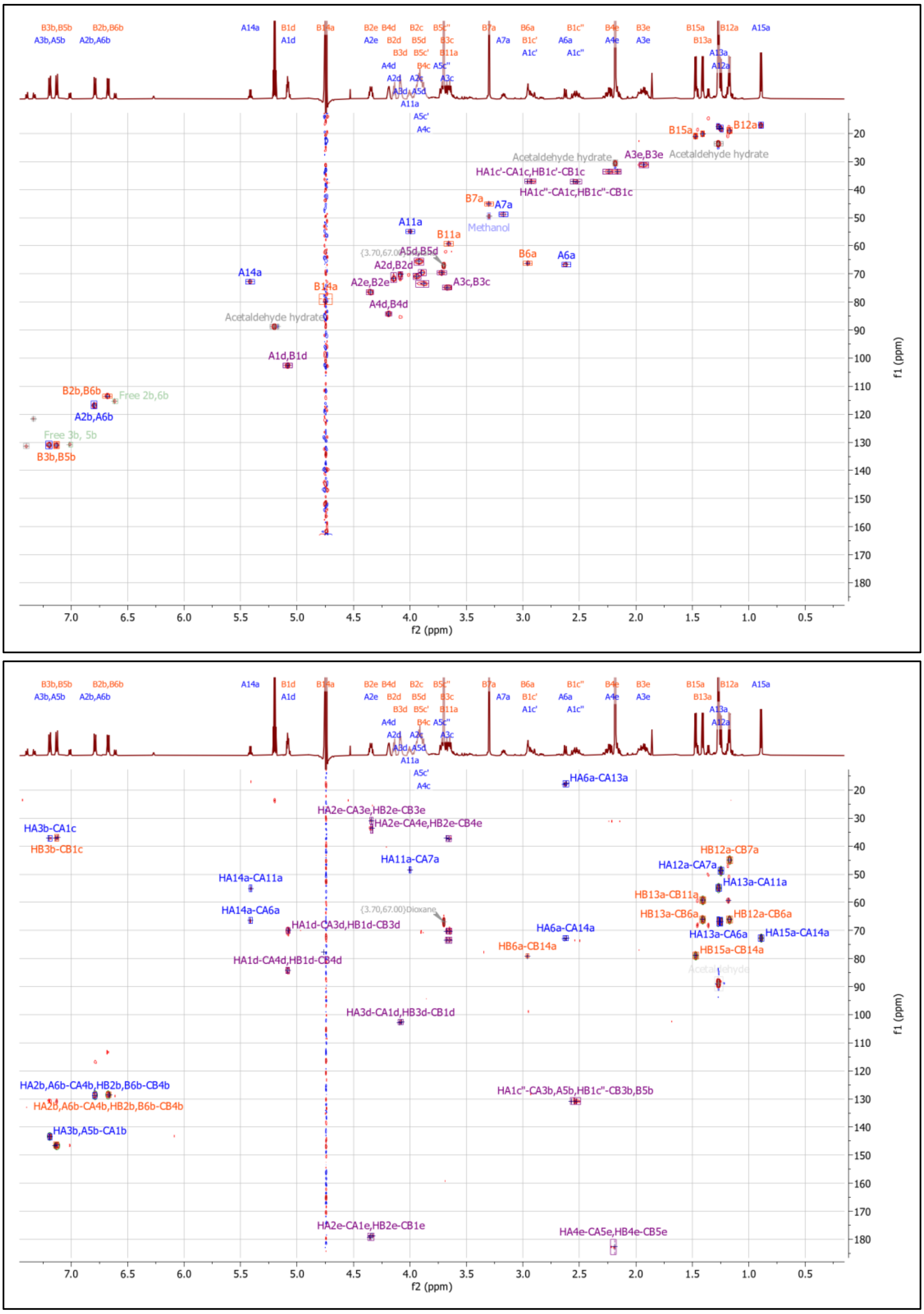

Figure S18: HSQC and HMBC spectra (whole spectrum)

HSQC (top) and HMBC (bottom) 2D NMR spectra of N^5,N^{10}-ethylene-H_4MPT diastereoisomers A (blue labels), B (orange labels) and overlapping signals (purple). f1 trace corresponds to the ^{1}H shown in Figure S17.

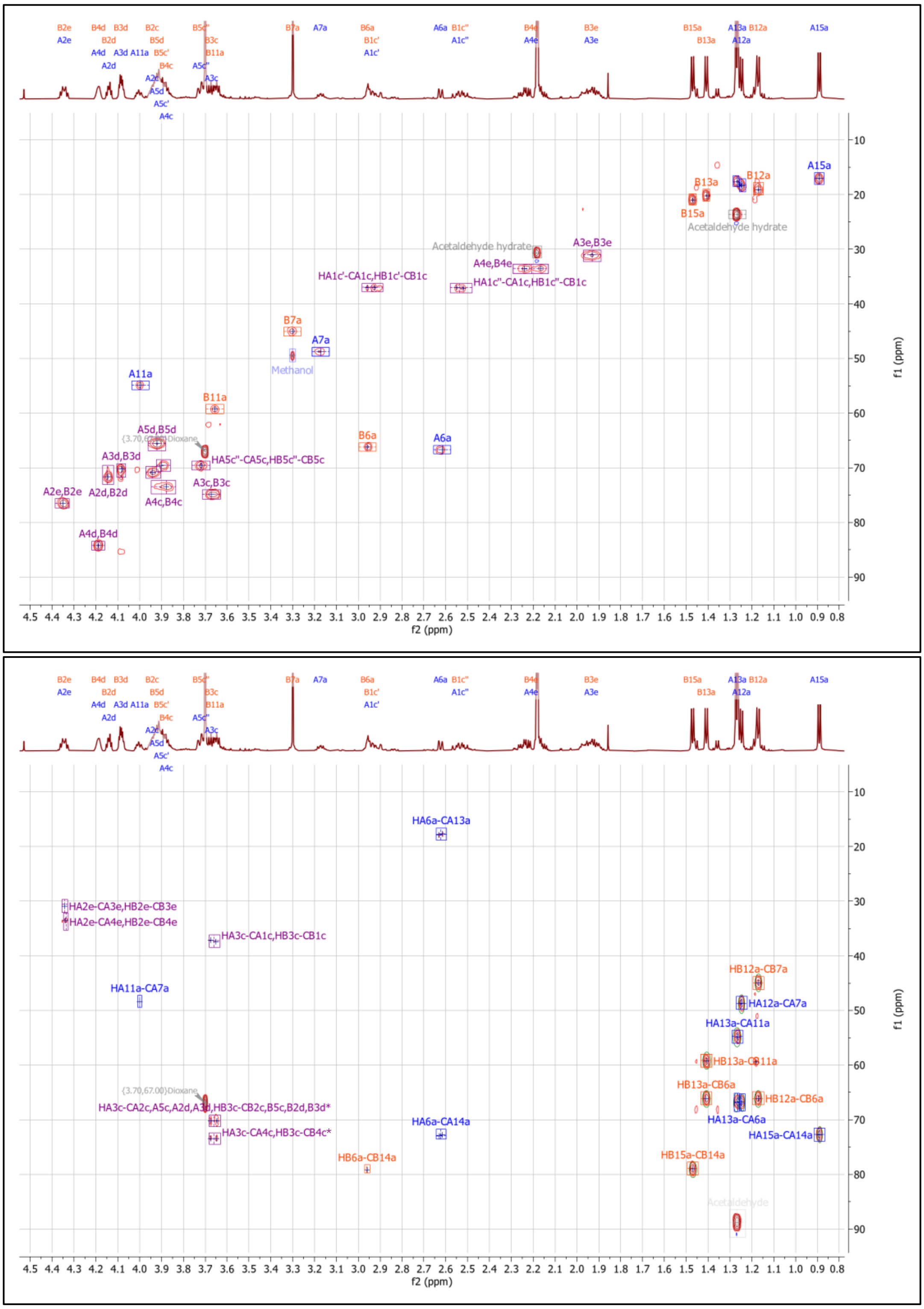

Figure S19: HSQC and HMBC spectra (expansion)

Expansion [10-90 ppm for ^{13}C and 0.8-4.4 ppm for ^{1}H] of the HSQC (top) and HMBC (bottom) 2D NMR spectra. Diastereoisomer A is in blue labels, isomer B in orange labels. Signals that are indistinguishable are labelled in purple. f1 trace corresponds to the ^{1}H spectra shown in Figure S17.

n°	Name	f1 Shift	f2 Shift	f1 Range	f2 Range
1	Methenyl 2b,6b	131.29	7.397	130.340 .. 132.190	7.422 .. 7.374
2	A3b,A5b	130.96	7.194	129.541 .. 132.214	7.226 .. 7.169
3	B3b,B5b	130.96	7.129	129.714 .. 132.204	7.155 .. 7.106
4	Free 3b, 5b	130.8	7.013	129.977 .. 131.628	7.023 .. 6.988
5	Methenyl 3b,5b	121.62	7.334	120.812 .. 122.397	7.347 .. 7.318
6	A2b,A6b	116.78	6.798	115.255 .. 117.955	6.824 .. 6.771
7	Free 2b,6b	115.33	6.616	114.507 .. 116.158	6.631 .. 6.588
8	B2b,B6b	113.56	6.68	112.734 .. 114.385	6.722 .. 6.639
9	A1d,B1d	102.6	5.083	101.776 .. 103.427	5.124 .. 5.042
10	Acetaldehyde hydrate	88.743	5.196	87.952 .. 89.534	5.239 .. 5.160
11	A4d,B4d	84.231	4.189	83.405 .. 85.056	4.219 .. 4.160
12	B14a	78.92	4.748	77.164 .. 80.957	4.803 .. 4.689
13	A2e,B2e	76.495	4.35	75.670 .. 77.321	4.386 .. 4.322
14	A3c,B3c	74.884	3.671	74.059 .. 75.709	3.713 .. 3.630
15	A4c,B4c	73.434	3.878	72.277 .. 74.763	3.948 .. 3.834
16	A14a	72.789	5.417	71.998 .. 73.580	5.456 .. 5.378
17	A2d,B2d	71.661	4.146	69.544 .. 73.016	4.169 .. 4.119
18	A2c,B2c	70.855	3.943	69.688 .. 71.948	3.971 .. 3.904
19	A3d,B3d	70.211	4.082	69.236 .. 71.722	4.104 .. 4.064
20	HA5c"-CA5c,HB5c"-CB5c	69.566	3.719	68.741 .. 70.391	3.760 .. 3.678
21	HA5c'-CA5c,HB5c'-CB5c	69.566	3.9	68.476 .. 70.654	3.924 .. 3.858
22	A6a	66.665	2.615	65.874 .. 67.457	2.655 .. 2.576
23	B6a	66.182	2.958	65.391 .. 66.973	2.997 .. 2.918
24	A5d,B5d	65.537	3.921	64.367 .. 66.873	3.962 .. 3.881
25	B11a	59.253	3.656	58.427 .. 60.078	3.697 .. 3.615
26	A11a	54.9	3.996	54.110 .. 55.693	4.036 .. 3.957
27	Methanol	49.422	3.3	48.243 .. 50.614	3.314 .. 3.287
28	A7a	48.778	3.172	47.987 .. 49.569	3.211 .. 3.133
29	B7a	45.071	3.3	44.280 .. 45.863	3.340 .. 3.261
30	HA1c"-CA1c,HB1c"-CB1c	37.095	2.535	36.276 .. 37.966	2.580 .. 2.480
31	HA1c'-CA1c,HB1c'-CB1c	37.014	2.938	36.223 .. 37.805	2.983 .. 2.886
32	A4e,B4e	33.555	2.206	32.750 .. 34.458	2.292 .. 2.130
33	A3e,B3e	31.051	1.93	30.130 .. 32.236	1.971 .. 1.889
34	Acetaldehyde hydrate	30.729	2.187	29.423 .. 31.581	2.203 .. 2.162
35	Acetaldehyde hydrate	23.639	1.266	22.847 .. 24.430	1.306 .. 1.227
36	B15a	21.091	1.474	19.875 .. 21.913	1.487 .. 1.455
37	B13a	20.254	1.405	19.050 .. 21.186	1.424 .. 1.393
38	B12a	19.126	1.17	17.777 .. 20.095	1.193 .. 1.146
39	A12a	18.241	1.25	17.106 .. 19.450	1.256 .. 1.228
40	A13a	17.676	1.266	16.519 .. 18.545	1.284 .. 1.259
41	A15a	17.031	0.892	16.005 .. 18.141	0.914 .. 0.870

Figure S20: Assignment table for HSQC NMR spectrum

Assignments for the HSQC spectrum of N^5,N^{10}-ethylene-H_4MPT (isomer A and B) from Figure S18 and S19.

n°	Name	f1 Shift	f2 Shift	f1 Range	f2 Range
1	Acetaldehyde	88.804	1.268	86.446 .. 91.528	1.302 .. 1.229
2	HA11a-CA7a	48.409	4	47.234 .. 49.446	4.008 .. 3.991
3	HA12a-CA6a	67.099	1.247	65.244 .. 68.359	1.256 .. 1.230
4	HA12a-CA7a	48.736	1.248	47.461 .. 49.816	1.272 .. 1.221
5	HA13a-CA11a	54.836	1.265	53.658 .. 56.013	1.291 .. 1.240
6	HA13a-CA6a	66.848	1.264	65.147 .. 68.455	1.281 .. 1.257
7	HA14a-CA11a	55.065	5.41	53.837 .. 56.220	5.421 .. 5.401
8	HA14a-CA6a	66.435	5.412	65.342 .. 67.528	5.426 .. 5.397
9	HA15a-CA14a	72.738	0.891	71.560 .. 73.916	0.916 .. 0.865
10	HA1c"-C(A3b,A5b),HB1c"-C(B3b,B5b)	130.81	2.542	129.561 .. 131.946	2.579 .. 2.490
11	HA1d-CA3d,HB1d-CB3d	69.984	5.078	68.920 .. 71.047	5.097 .. 5.060
12	HA1d-CA4d,HB1d-CB4d	84.214	5.083	83.150 .. 85.277	5.101 .. 5.065
13	H(A2b,A6b)-CA4b,H(B2b,B6b)-CB4b	128.74	6.789	127.676 .. 129.803	6.807 .. 6.771
14	H(A2b,A6b)-CA4b,H(B2b,B6b)-CB4b	128.51	6.668	127.447 .. 129.573	6.691 .. 6.648
15	HA2e-CA1e,HB2e-CB1e	178.93	4.345	177.765 .. 180.297	4.372 .. 4.320
16	HA2e-CA3e,HB2e-CB3e	30.601	4.343	29.728 .. 32.105	4.356 .. 4.330
17	HA2e-CA4e,HB2e-CB4e	33.338	4.337	32.195 .. 35.323	4.349 .. 4.327
18	H(A3b,A5b)-CA1b	143.43	7.19	142.365 .. 144.492	7.209 .. 7.172
19	H(A3b,A5b)-CA1b,H(B3b,B5b)-CB1b	146.51	7.133	145.349 .. 147.608	7.154 .. 7.109
20	HA3b-CA1c	37.163	7.196	36.171 .. 38.179	7.205 .. 7.171
21	HA3c-CA1c,HB3c-CB1c	37.296	3.661	36.193 .. 38.456	3.683 .. 3.633
22	HA3c-C(A2c/A5c/A2d/A3d),HB3c-C(B2c/B5c/B2d/B3d)*	70.213	3.659	69.150 .. 71.277	3.682 .. 3.629
23	HA3c-CA4c,HB3c-CB4c*	73.426	3.66	72.363 .. 74.490	3.683 .. 3.630
24	HA3d-CA1d,HB3d-CB1d	102.68	4.082	101.729 .. 103.487	4.106 .. 4.060
25	HA4e-CA5e,HB4e-CB5e	182.68	2.186	179.964 .. 185.403	2.226 .. 2.176
26	HA6a-CA13a	17.767	2.623	16.591 .. 18.869	2.644 .. 2.597
27	HA6a-CA14a	72.855	2.622	71.714 .. 73.564	2.645 .. 2.599
28	HB12a-CB6a	66.096	1.169	64.904 .. 67.260	1.196 .. 1.145
29	HB12a-CB7a	44.967	1.17	43.789 .. 46.144	1.196 .. 1.145
30	HB13a-CB11a	59.197	1.408	58.019 .. 60.374	1.434 .. 1.383
31	HB13a-CB6a	66.082	1.409	64.904 .. 67.260	1.434 .. 1.383
32	HB15a-CB14a	78.935	1.47	77.757 .. 80.112	1.495 .. 1.444
33	HB3b-CB1c	37.04	7.128	35.708 .. 38.140	7.148 .. 7.110
34	HB6a-CB14a	79.164	2.96	78.221 .. 79.720	2.972 .. 2.948

Figure S21: Assignment table for HMBC NMR spectrum

Assignments for the HMBC spectrum of N^5,N^{10}-ethylene-H_4MPT (isomer A in blue, isomer B in orange) from Figure S18 and S19. *: uncertain assignments; /: uncertain carbon shift attribution. NB: Complex peak names from the spectra were renamed with additional characters for better readability.

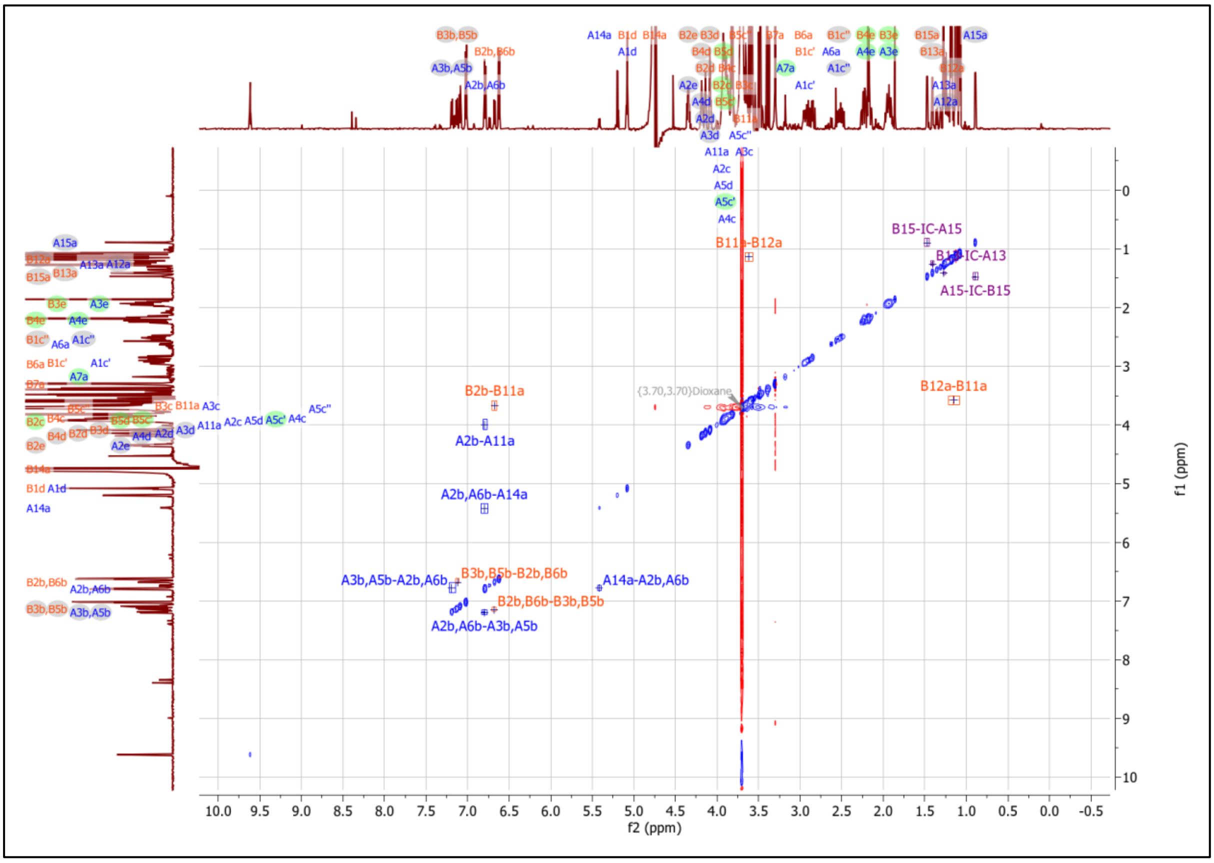

Figure S22: EASY-ROESY NMR spectrum

EASY-ROESY NMR spectrum of a mixture of *N*[5],*N*[10]-ethylene-H_4MPT, H_4MPT, and acetaldehyde. Cross peaks for *N*[5],*N*[10]-ethylene-H_4MPT diastereoisomers A (blue labels), B (orange labels) and isomer interconversion peaks (purple) are highlighted. Assignments on the ^{1}H trace have been transposed from the ^{1}H spectrum in figure S17.

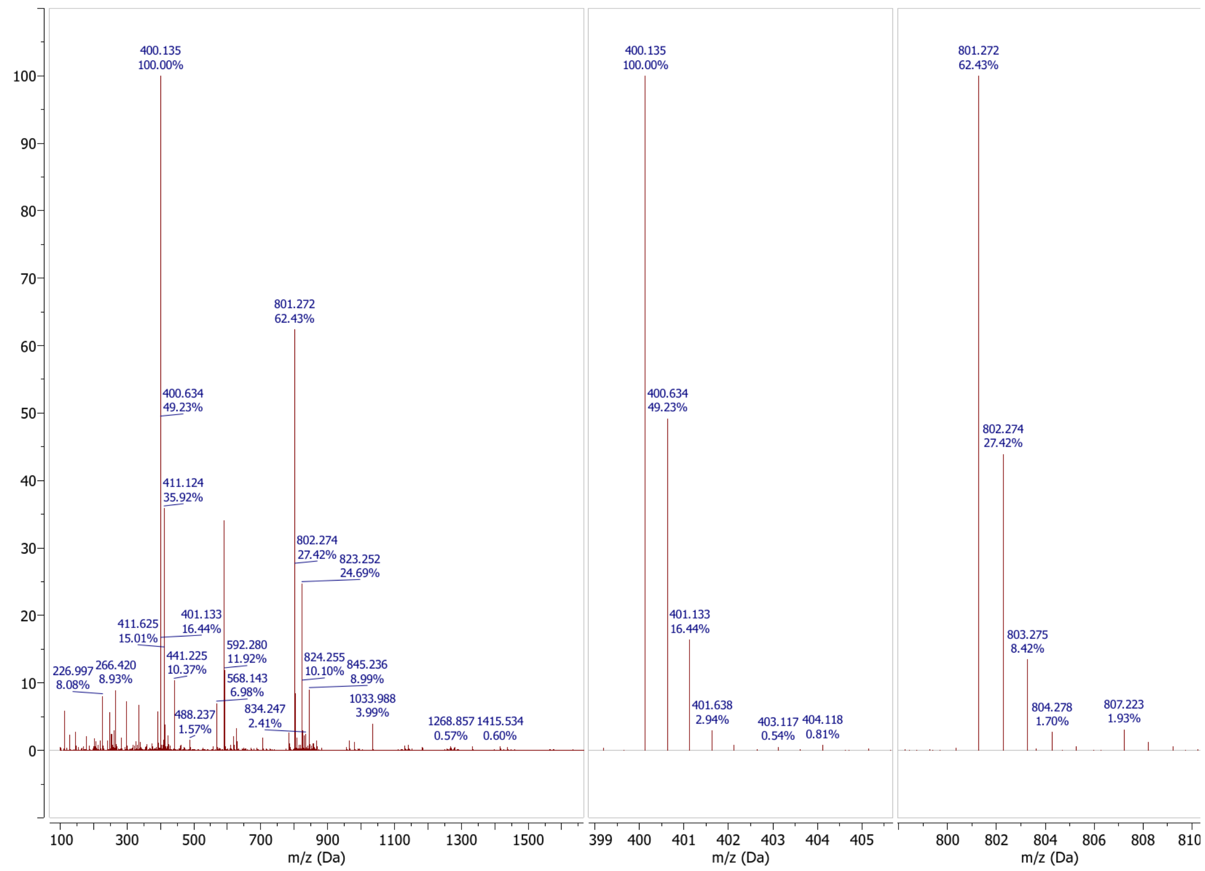

Figure S23: MS-QTOF spectrum

A MS centroid spectrum of N^5,N^{10}-ethylene-H_4MPT (MS-QTOF, ESI negative). Left: Global spectrum; Center and right: Zoom on main ion peaks at 400.135 and 801.272 m/z corresponding to N^5,N^{10}-ethylene-H_4MPT.

II – MATERIALS AND METHODS

1 – Methodology for anaerobic conditions

Experiment procedures described as conducted in “anaerobic conditions” were performed in an anaerobic chamber (95% N_2 / 5% H_2), using oxygen free labware and solutions. Solutions were made anoxic by sparging with N_2 for 1 hour at 2 SLPM under stirring, and labware by remaining open in anaerobic chamber atmosphere for 24h for glassware and 72h for plasticware. Steps of anaerobic processes that could not be performed in anaerobic tents were carried out by limiting oxygen exposure:

- Evaporation: To prevent oxygen exposure during transfers between anaerobic chamber and evaporator, the airtight samples were rapidly opened and placed under vacuum. For removal, the vacuum was replaced with N_2 atmosphere, and the samples were quickly closed back airtight under a slight flow N_2 before being transferred back to the anaerobic chamber.
- Cold storage: Solutions or dried extracts were stored in anoxic glass vials under 100% N_2 gas phase, with 0.1 bar overpressure.
- Ultracentrifugation: ultracentrifuge tubes were made anoxic (see anoxic plasticware above) and transferred between the centrifuge and the anaerobic chamber with an O-ring-sealed lid.
- NMR measurements: Samples measured only for short ^{1}H-NMR (<=32scans, single timepoint) were measured in standard NMR tubes while samples requiring longer measurement (>32 scans ^{1}H, multi-point ^{1}H, ^{13}C, HSQC, HMBC, EASY-ROESY) were measured in NMR tubes equipped with Young valves to ensure anoxicity (see section II-11).

2 – Cultivation of Methanothermobacter marburgensis

Methanothermobacter marburgensis (DSM 2133) was cultivated at +65°C and 600 rpm stirring in a Sartorius Biostat C-plus10 L bioreactor (Typ C10-3) within a working volume of 9 L. The cultures were fed with a constant gas flow of 2 SLPM 72% H_2 / 18% CO_2 / 10% (1% H_2S in N_2). Cultures were maintained in the laboratory for several generations, with each culture inoculated using 200 mL of a previous culture, which had been stored anaerobically in 1 L bottles at 7 °C. The growth medium, as described by Duin et al. 2011[2], consisted of 51.7 mM KH_2PO_4, 50.5 mM NH_4Cl, 31.3 mM Na_2CO_3, and trace elements (329 µM $MgCl_2{\cdot}6H_2O$, 83 µM $FeCl_2{\cdot}4H_2O$, 1.4 µM $CoCl_2{\cdot}6H_2O$, 1.38 µM $MoO_4{\cdot}2H_2O$, and 8.4 µM $NiCl_2{\cdot}6H_2O$ complexed in 262 µM nitrilotriacetic acid), and was made anoxic prior to inoculation by sparging with 100% N_2 at 5 SLPM for 1 h. The gas mixes were externally controlled via AliCat flow controllers from individual gas bottles (AGA, Finland) of H_2 (grade 5.0), CO_2 (grade “anaerobe”, 4.0) and H_2S (custom-made mixture of 1.0% in N_2). The cells were harvested after 24-36 h of culture ($OD_{600} \geq 7$).

3 – Cell harvesting

Prior to harvesting, cells were either kept under the growth gas mix while being cooled down to +4°C (referred to as cells “harvested under standard conditions”), or sparged with 100% H_2 (2 SLPM) 2h before being cooled down to +4°C under this gas mix (referred to as cells “harvested under H2”). The cells were harvested by continuous flow centrifugation using a Sorvall Contifuge Stratos centrifuge with a titanium rotor (Multifuge X1R TX-200) that was operated at

17000 rpm and 4 °C. Harvesting tubing connecting the bioreactor to the rotor was sparged with 0.5 SLPM of N_2 for at least an hour before harvesting. The biomass was harvested anaerobically at approx. 100-200 mL/min by creating a slight overpressure inside the bioreactor gas phase, either with 100% N_2 (for cells "harvested under standard conditions") or H_2 (for cells "harvested under H_2"). After centrifugation, the tubing was closed, and the rotor transferred into the anaerobic chamber, in which the biomass was retrieved. The collected wet biomass (approx. 100-150 g) was transferred into 500 mL bottles and stored at -20°C until used.

<u>4 – Cell lysate preparations</u>

These procedures were performed in anaerobic conditions (see section II-1). Two different methods were used for the production of cell lysate. With the first method (sonication method), biomass cell lysis was carried out by resuspending 100-150 g of unfrozen wet biomass in Milli-Q water at a 1:1 ratio (v/w) in a Sonopuls rosette cell, then sonicating the suspension with a Bandelin Sonopuls Homogenizer HD 3200 equipped with a VS70 T probe. The sonication sequence consisted of 6 rounds of 5 minutes (0.5 s pulse / 0.5 s rest) at 87% power with gentle stirring between each round. For cell lysate experiments, aliquots of 5 mL of the resulting solution (referred to as "*M. marburgensis* sonicated cells lysate") were transferred into 50 mL anoxic sealed serum vials and stored at -20°C with N_2 overpressure until used in downstream experiments.

With the second method (CTAB method), cell permeabilization for H_4MPT purification was performed by resuspending 100-150g of unfrozen wet biomass in MOPS/NaOH buffer 50mM pH 7 at a 1:1 ratio (v/w) and mixing the suspension previously heated at 60°C with prewarmed Cetyltrimethylammonium bromide (CTAB) to a final concentration of 1%. The resulting cell suspension was then transferred in an airtight vial and maintained at 60°C under gentle shaking outside of the anaerobic chamber for exactly 6 min before being cooled on ice. The resulting suspension (referred to as "*M. marburgensis* CTAB cell extracts") was directly used for pterin purification procedures.

<u>5 – Purification of H_4MPT and N^5-Ethyl-H_4MPT from *M. marburgensis* biomass</u>

This procedure was performed in anaerobic conditions (see section II-1). Free H_4MPT was obtained from CTAB cell extracts prepared from cells harvested in standard conditions. Mixtures of N^5-ethyl-H_4MPT and free H_4MPT could be obtained from both CTAB cell extracts and sonicated cells lysates when prepared from cells harvested under H_2 but higher concentrations of N^5-ethyl-H_4MPT were obtained from sonicated cells lysate preparations. The purification protocol for the retrieval of H_4MPT and N^5-ethyl-H_4MPT was adapted from the methods described in Breitung et al. 1992[3]. Sonicated cells lysates or CTAB cell extracts were ultra-centrifuged at 120 000 g for 40 min immediately after preparation. The obtained supernatant was adjusted to pH 3 with pure formic acid and stored for less than 12 hours at 4°C in the dark. The supernatant was then applied at 2.5 mL/min to a 160mL XAD-2 column prepared in a CHROMABOND® Flash DL shaft, previously washed with 500 mL of pure methanol, rinsed with 500 mL of water and equilibrated with 1 L of 1/70 Formic acid in water (v/v) (5 mL/min). After loading the supernatant, the column was washed at 2.5 mL/min with 400 mL of 1/70 formic acid in water (running solution, v/v) until no absorbance at 280nm could be detected in the flow through. Free H_4MPT and N^5-ethyl-H_4MPT were eluted using 15% methanol at the same flow rate and collected in 8mL fractions. Dilutions in 1M potassium phosphate buffer pH 7 were analyzed in UV spectrometry (the pH of each UV

sample was confirmed to be pH 7). Fractions showing absorbance extrema between 290 and 302 nm were pooled and evaporated in rotary evaporator until the global pool volume is reduced by at least 15%. The product was stored for less than 12 hours at 4°C in the dark.

The resulting solution was diluted at a 1:1.5 ratio (v/v) with 50 mM MOPS/NaOH pH 7, adjusted to pH 7 with 1 M NaOH then applied to a DEAE-Sephadex A25 column prepared in a Cytiva XK 26/20 shaft (50mL of final column volume, packed on 1 M NaCl) previously equilibrated with 3 column volumes of 50 mM MOPS /NaOH Buffer pH 7 (running buffer) at 2.5 mL/min. After loading the extract at the same flow rate, the column was washed with at least 3 column volumes of running buffer until stabilisation of the flow through absorbance at 280 nm. *N*^{5}-ethyl-H_4MPT and free H_4MPT were eluted by running at least 3 column volumes of 2M NaCl running buffer. The collected fractions were analysed in UV spectrometry and pooled using the same criteria as for the XAD elution fractions. The pool was then desalted and concentrated on a C18 cartridge (see section II-6).

<u>6 – Pterin desalting/concentration</u>

This procedure was performed in anaerobic conditions (see II-1). The pterin containing solutions were adjusted to pH 3 with 100 % formic acid if needed then loaded on a Waters Sep-Pak C18 Plus Short Cartridge 360 mg that was previously washed with 20 mL of pure methanol, rinsed with 20 mL of water and equilibrated with 20mL of 1/70 Formic acid in water (v/v) (running solution). All liquids were run on the column at a dropwise flow rate. The column was then washed with 20 mL of running solution. For H_4MPT or *N*^{5}-ethyl-H_4MPT batch purification, the compounds were eluted with 20 mL of 10% Methanol in running solution while for global pterin extraction, the column was first dried with chamber atmosphere before eluting the compounds with 20 mL of 100% methanol. The resulting solution was then evaporated with a rotary evaporator until visually dry. For eventual storage, the dried compounds were resuspended in water, aliquoted in small vials, evaporated again and stored at -20°C in anaerobic conditions in dark until use.

<u>7 – Synthesis of the C1-carrying H_4MPT forms (*N*^{5},*N*^{10}-methylene- and *N*^{5}-methyl-) from free H_4MPT</u>

These procedures were performed in anaerobic conditions (see section II-1). Synthesis of *N*^{5},*N*^{10}-methylene-H_4MPT by condensation of formaldehyde on free H_4MPT and its reduction by potassium borohydride salts was performed following the adapted protocol established in Keltjens et al. 1986.[4] Formaldehyde was prepared anaerobically by heating paraformaldehyde in a 1 mM NaOH solution at 65°C for 60 min with vigorous shaking, then cooled down and adjusted to pH 7 with 1M HCl before use. *N*^{5},*N*^{10}-*m*ethylene-H_4MPT was formed by resuspending 0.1-15 µmol of free H_4MPT in 500 µL of 25mM potassium phosphate buffer pH 7 with 5 molar equivalents of formaldehyde and incubating 15 min at room temperature. NMR (600 MHz, see section II-11) and UV analysis were eventually performed on the resulting solution. *N*^{5}-*m*ethyl-H_4MPT was formed by adding an excess of KBH_4 to the previous solution and incubating at 60°C over 10 min. The solution was then acidified to pH <3 with 1 M HCl and desalted on a C18 cartridge (see section II-6), then adjusted to pH 7 with NaOH and measured in NMR at 600 MHz (see section II-11).

8 – Synthesis of the C2-carrying H_4MPT forms (*N*^{5},*N*^{10}-ethylene-, *N*^{5}-ethyl-) from free H_4MPT:

These procedures were performed in anaerobic conditions (see section II-1). *N*^{5},*N*^{10}-ethylene-H_4MPT synthesis and analysis was performed identically to *N*^{5},*N*^{10}-*m*ethylene-H_4MPT but using 100 equivalents of acetaldehyde and incubating at 60min at 30°C. Acetaldehyde was prepared on ice by diluting pure acetaldehyde to water (1/5 v/v) and degassed using freeze-pump-thaw method (4 cycles). The quality/concentration was confirmed via NMR measurement weekly. *N*^{5}-ethyl-H_4MPT synthesis and analysis were performed identically to *N*^{5}-methyl-H_4MPT, with an *N*^{5},*N*^{10}-ethylene-H_4MPT starting solution.

9 – *In vitro* cell lysate experiments

Anaerobically-stored serum vials containing 5 mL of *M. marburgensis* sonicated cells lysate were thawed at room temperature and sparged with 100% N_2 or H_2 at a flow rate of 1.5 SLPM for 1.5 hours and 200 rpm stirring, except for "No Treatment" that was only briefly (3 seconds) flushed with N_2. Acetaldehyde or ^{13}C-labeled acetaldehyde was added anaerobically to the relevant vials to a final concentration of 3 mM. The vials were briefly flushed with the corresponding gas to remove potential contaminants from the tent atmosphere and incubated at +65°C (200 rpm stirring) for two hours. The following steps were performed in anaerobic conditions (see section II-1). Lysates were transferred into 15 mL Falcon tubes and pH-adjusted to below 3 using 100% formic acid, followed by centrifugation at 6500 × g for one hour. Supernatants were desalted and concentrated on C18 cartridges (see section II-6). Dried samples were then resuspended in 500 µL of water, adjusted to pH 7 using NaOH or HCl, and measured in ^{1}H-NMR at 600 MHz and quantified (see section II-11).

10 – Spontaneous *N*^{5},*N*^{10}-ethylene-H_4MPT reduction assay

1 mL solution of 0.25 mM H_4MPT, 25 mM acetaldehyde and 100mM potassium phosphate buffer pH7 (all compounds being oxygen free) was incubated in 10mL anoxic sealed glass vials with 100% H_2 gas phase at 65°C for 2h at 200rpm shaking (n=3). The solution was then measured in ^{1}H-NMR at 600 MHz and quantified (see section II-11).

11 – Single timepoint NMR data acquisition (cell lysate assay, spontaneous *N*^{5},*N*^{10}-ethylene-H_4MPT reduction assay, pterin characterization)

NMR experiments were performed in 100mM potassium phosphate buffer pH7 with 10% D_2O at 298 K. Chemical shifts were referenced to 1,4-dioxane at 3.7 ppm (^{1}H) and 67 ppm (^{13}C). The spectra were acquired on a Bruker Avance NEO 600 spectrometer equipped with a 5 mm He-cooled TCI cryoprobe operating at 600.13 and 150.90 MHz for ^{1}H and ^{13}C, respectively, using TopSpin 4.1.3, 4.4.1 or 4.5.0 software. Data acquisition was done. All ^{1}H detected 1D and 2D experiments were performed using pulse sequences with water suppression (presaturation and/or excitation sculpting). ^{1}H experiments were performed using 1D NOESY pulse sequence with presaturation during relaxation delay and mixing time (pulse program: *noesygppr1d*). Spectra were acquired with 12 s relaxation delay, 32, 64 or 128 transients (128 transients were exclusively used for pterin characterization when 2D spectra were also measured), a spectral width of 16 ppm, and 64 K data points. ^{1}H,^{1}H EASY-ROESY experiments were performed with excitation sculpting using gradients and presaturation during the relaxation delay (in-house pulse program based on *roesyadesjsph*). EASY-ROESY spectra were acquired with spectral width of 11

ppm in both dimensions using 2 s relaxation delay, 2 K data points, 128-256 t_1 increments, and 16-32 transients. ROESY spinlock time was 250 ms. ^{1}H,^{13}C HSQC experiments were performed with presaturation during relaxation delay (pulse program: *hsqcetgpprsisp2.2*). HSQC spectra were acquired with spectral widths of 11 ppm for ^{1}H and 165 ppm for ^{13}C using 2 s relaxation delay, 2 K data points, 256 t_1 increments, and 16 or 32 transients. ^{1}H,^{13}C HMBC experiments were performed with additional gradients to reduce effects of radiation damping and presaturation during relaxation delay (in-house pulse program based on *hmbcetgpl2nd*). HMBC spectra were acquired with spectral widths of 11 ppm for ^{1}H and 235 ppm for ^{13}C using 2 s relaxation delay, 4 K data points, 128-256 t_1 increments, and 24, 48 or 64 transients. For ^{13}C spectra, uniform driven equilibrium Fourier transform (UDEFT) experiment with power-gated decoupling was used (pulse program: *udeft_pg*). UDEFT spectra were acquired with 4 s relaxation delay, 5 K transients, a spectral width of 235 ppm, and 25714 data points.

12 – NMR data processing for relative quantification (*in vitro* cell lysate experiments, spontaneous *N*^{5},*N*^{10}-ethylene-H_4MPT reduction assay)

Relative quantitative values for each species were deduced from integration values of species-specific peaks isolated from eventual overlaps by spectrum deconvolution, except for the quantitative reference compound peak (dioxane) which was directly integrated. The different species were identified based on the specific shift of the 2 pairs of doublets corresponding to their aromatic protons in the range 6.5-7.3 ppm. Free H_4MPT (6.60 ppm, 7.01 ppm), *N*^{5},*N*^{10}-ethylene-H_4MPT isomer A (6.67 ppm, 7.13 ppm) and B (6.80 ppm, 7.19 ppm), *N*^{5}-ethyl-H_4MPT (6.77 ppm, 7.13 ppm) could be identified along with 3 additional unknown compounds named X (6.77 ppm, 7.09 ppm), Y (6.94 ppm, 7.16 ppm) and Z (6.72ppm, 7.07 ppm). "Clean peaks" that were not overlapping with any other signals could be defined for Free H_4MPT (7.01 ppm), *N*^{5},*N*^{10}-ethylene-H_4MPT (7.19 and 6.67 ppm), X (7.09 ppm), Y (6.94 ppm) and Z (6.72ppm) and thus were used for quantification. For *N*^{5}-ethyl-H_4MPT, integration values were deduced from the integration value of the overlaps that include *N*^{5}-ethyl-H_4MPT peaks to which were subtracted the integration values of the clean peaks identified for the overlapping species.

Relative quantitative values were defined as the integration value of the clean characteristic peak for Free H_4MPT, X, Y and Z, as the sum of clean peaks integration value for *N*^{5},*N*^{10}-ethylene-H_4MPT (concentration of each isomer being considered equal, identical to the average of isomer concentrations multiplied by 2) and as the average of the two deduced integration values for *N*^{5}-ethyl-H_4MPT. These relative values were normalized among experiments with their respective experiment dioxane values. Relative values that appeared negative (V_{neg}) were replaced by the value below:

$$V_{neg} = \frac{LOD}{\sqrt{2}} \qquad (1)$$

where LOD is the limit of detection, arbitrarily attributed here to the order of magnitude of the lowest measured integration value (10^{11})[5].

For each replicate, the values for free H_4MPT, *N*^{5},*N*^{10}-ethylene-H_4MPT and *N*^{5}-ethyl-H_4MPT were converted to percentages of identified pterins, with an associated standard deviation for *N*^{5},*N*^{10}-ethylene-H_4MPT and *N*^{5}-ethyl-H_4MPT (due to the averaging of different integration values, and the propagation of uncertainty) calculated as follows:

$$SD_{\%} = V_{\%} \cdot \sqrt{\left(\frac{SD}{V}\right)^2 + \left(\frac{\sum_{i=1}^{n} SD_i}{\sum_{i=1}^{m} V_i}\right)^2} \quad (2)$$

Where SD is the relative standard deviation, V the value associated with the standard deviation, n the number of different standard deviations taken into account for the percentage calculation, and m the number of different standard deviations taken into account for the percentage calculation. The percentage values were averaged across replicates. Standard deviations were calculated across single values per replicate for free H_4MPT and as the root mean square of the existing SDs per replicates for *N*^{5},*N*^{10}-ethylene-H_4MPT and *N*^{5}-ethyl-H_4MPT. Intra and inter experiments statistical analyses were performed using a one-way ANOVA followed by Tukey's HSD post hoc test in R version 2025.5.1.0 (Build 513).

13 – NMR spectra simulation

^{1}H-NMR spectra simulations were performed on iNMR 6.5.0. The simulated spectrum was overlayed with the measured spectra in order to derive the coupling constants for the ethyl group within *N*^{5}-ethyl-H_4MPT.

14 – Quantification of free H_4MPT and ethylene-H_4MPT for kinetic studies (acetaldehyde condensation) in UV-Vis

This procedure was performed in anaerobic conditions (see section II-1). Acetaldehyde condensation on free H_4MPT was followed in UV spectrometry by adding acetaldehyde to a solution of H_4MPT in potassium phosphate buffer pH 7. The reactions were started by adding 500µL of acetaldehyde solution to 500µL in H_4MPT with buffer. The targeted concentrations at t_0 were 0.05 mM H_4MPT, 100mM potassium phosphate buffer pH 7 and 5 mM, 2 mM,1 mM or 0.5mM of acetaldehyde, respectively. UV spectra were measured every 5 seconds over 60min at 25°C. Concentrations of free H_4MPT and *N*^{5},*N*^{10}-ethylene-H_4MPT over time were calculated using the formula below.[6]

$$C_1 = \frac{A^{\lambda_2}\varepsilon_2^{\lambda_1} - A^{\lambda_1}\varepsilon_2^{\lambda_2}}{\varepsilon_1^{\lambda_2}\varepsilon_2^{\lambda_1} - \varepsilon_1^{\lambda_1}\varepsilon_2^{\lambda_2}} \quad (3)$$

$$C_2 = \frac{A^{\lambda_1}\varepsilon_1^{\lambda_2} - A^{\lambda_2}\varepsilon_1^{\lambda_1}}{\varepsilon_1^{\lambda_2}\varepsilon_2^{\lambda_1} - \varepsilon_1^{\lambda_1}\varepsilon_2^{\lambda_2}} \quad (4)$$

with C_1 and C_2 the concentrations of free H_4MPT and *N*^{5},*N*^{10}-ethylene-H_4MPT in mM respectively, λ_1 and λ_2 the wavelength where the difference between H_4MPT and *N*^{5},*N*^{10}-ethylene-H_4MPT UV spectra are maximal (268 nm and 316 nm respectively) and $\varepsilon_1^{\lambda_1}$, $\varepsilon_1^{\lambda_2}$, $\varepsilon_2^{\lambda_1}$, $\varepsilon_2^{\lambda_2}$ the epsilon molar of free H_4MPT and *N*^{5},*N*^{10}-ethylene-H_4MPT at λ_1 and λ_2 obtained from our UV standard spectra for both species ($\varepsilon_1^{\lambda_1}$=8.70 mM^{-1}.cm^{-1}, $\varepsilon_1^{\lambda_2}$=9.42 mM^{-1}.cm^{-1}, $\varepsilon_2^{\lambda_1}$ =13.90 mM^{-1}.cm^{-1}, $\varepsilon_2^{\lambda_2}$=5.45 mM^{-1}.cm^{-1}).

15 – Quantification of free H_4MPT and *N^5,N^{10}*-ethylene-H_4MPT for kinetic studies (*N^5,N^{10}*-ethylene-H_4MPT hydrolysis) through multiple timepoint NMR spectra acquisition

This procedure was performed in anaerobic conditions (see II-1). *N^5,N^{10}*-ethylene-H4MPT hydrolysis was measured by mixing 0.1mM Acetaldehyde with 0.2mM H4MPT (molar ratio of 1:2, n=1) or 0.4mM Acetaldehyde with 0.5mM H_4MPT (molar ratio of 1:0.8, n=2) in a solution of 50mM potassium phosphate pH 7 and 0.195 mM dioxane (300μL final volume) and incubating at room temperature over 120 min before diluting the sample with 300μL of water and immediately measuring the sample in NMR. ^{1}H spectra were acquired using 1D NOESY pulse sequence with presaturation during relaxation delay and mixing time (pulse program: *noesygppr1d*). Spectra were then acquired for a duration of 200 min to 570 min at 25°C with 12 s relaxation delay, 8 or 32 transients, a spectral width of 16 ppm, and 64 K data points.

NMR spectra were processed on MestReNova 15.0. The integration values were measured from direct signal integration after performing a multipoint baseline correction targeting specifically the signals to integrate: Free H_4MPT 6.60 ppm (d), 7.01 ppm (d); *N^5,N^{10}*-ethylene-H_4MPT isomer A 6.67 ppm (d), 7.13 ppm (d); *N^5,N^{10}*-ethylene-H_4MPT isomer B 6.80 ppm (d), 7.19 ppm (d); Acetaldehyde hydrate 5.20 ppm (q); Acetaldehyde non-hydrate 9.62 ppm (q). The related species concentrations per peak were calculated using the following formula:

$$C_{comp.} = \frac{I_{comp.}}{I_{ref.}} \times \frac{n_{ref.}}{n_{comp.}} \times C_{ref.} \qquad (5)$$

$C_{comp.}$ being the species concentration, $C_{ref.}$ the reference compound concentration (here, dioxane), $I_{comp.}$ and $I_{ref.}$ the integration value of the peaks of the compound to quantify (comp.) and the reference compound (ref.), $n_{comp.}$ and $n_{ref.}$ the number of protons corresponding to the measured signal of the compound to quantify (comp.) and the reference compound (ref.). The global concentrations were obtained by averaging the different concentrations per peak per compound. *N^5,N^{10}*-ethylene H_4MPT isomers concentrations were combined while acetaldehyde hydrate and non-hydrate were kept apart.

16 – Kinetic modelling of acetaldehyde condensation with free H_4MPT and *N^5,N^{10}*-ethylene-H_4MPT hydrolysis reactions: rate and equilibrium constants determination (CoPaSi)

The determined concentration of each quantified species at each timepoint (both condensation and hydrolysis assays) were used as input on CoPaSi v4.45 (Build 298). The biochemical model was defined as one fixed compartment with *N^5,N^{10}*-ethylene-H_4MPT, Free H_4MPT, Acetaldehyde hydrate and acetaldehyde non-hydrate as species with an equal weight of 1. Water was neglected from the reaction model since it is also the solvent of the reaction and is thus not taken into account for equilibrium constant calculations and is considered constant for kinetic parameters determination. Two reactions, "Hyd" (hydration) and "Cond" (condensation), with their respective rate constants Hyd.k1, Hyd.k2, Cond.k1 and Cond.k2, were set as follows:

$$CH_3CH(OH)_2 \underset{Hyd.k_2}{\overset{Hyd.k_1}{\rightleftarrows}} CH_3CHO \qquad \text{"Hyd"(6)}$$

$$CH_3CHO + H_4MPT \underset{Cond.k_2}{\overset{Cond.k_1}{\rightleftarrows}} CH_3CH{=}H_4MPT \qquad \text{"Cond"(7)}$$

For N^5,N^{10}-ethylene-H_4MPT hydrolysis (^{1}H-NMR-determined concentrations), only the datapoints acquired during the first 200 or 300 min of measurement were used for parameter estimation. Start values for kinetic constants were randomized between 10^{-6} and 10^6 $mM^{-1}\cdot s^{-1}$ or s^{-1} and start values for compound concentrations were randomized between 10^{-6} mM and 1 mM. For acetaldehyde condensation reactions (UV-determined concentrations), the data points measured from 30 to 1500 seconds of reaction were used for parameter estimation. Start values for "Cond" kinetic constants were randomized between 10^{-6} and 10^6 $mM^{-1}\cdot s^{-1}$ or s^{-1} while start values for "Hyd" kinetic constants were set by default to 10^6 s^{-1}. Start values for compound concentrations were randomized in an interval of their theoretical start concentration ±5%.

The parameter estimation was run using a genetic algorithm on a population of 1,000 elements over 10,000 generations. The output parameters were then used as inputs for time courses simulations of 10 hours reaction with 1 sec intervals. Equilibrium constants were calculated using the final concentrations of each compound (M) obtained from these timecourses as follows:

$$K_{eq}^{Condensation} = \frac{1}{K_{eq}^{Hydrolysis}} = \frac{[\mathrm{CH_3CH{=}H_4MPT}]}{[\mathrm{CH_3CHO}][\mathrm{H_4MPT}]} \quad (8)$$

The resulting estimated parameters were averaged alongside their standard deviation. High SD values were returned for estimations where some of the variables required for the reaction do not have experimental data, as it is the case Hyd.k1 in UV assays. However, the similarity of the values with their linear-regression-deduced equivalent confirmed the relevance of the estimated values, despite their high SD. A global corrected SD for the corresponding averages was calculated without considering each replicate individual SD returned during parameter estimation.

17 – MS-QTOF analysis

Samples were resuspended in a final 1:1 mixture of water and methanol and analyzed using a high-resolution mass spectrometer (6530 Accurate-Mass Q-TOF, Agilent Technologies, Santa Clara, USA). A high-performance liquid chromatograph (1260 Infinity HPLC, Agilent Technologies, Singapore) was used for sample injection without any column mounted on the system. Mass accuracy of the instrument using external calibration was specified to be ≤ 3ppm. Two injections (2 µL) were made for each sample with 100% acetonitrile at a flow rate of 0.25 $mL\cdot min^{-1}$. Data was acquired using Agilent MassHunter Data Acquisition (LC/MS), and processed with MassHunter Qualitative Analysis, version B.07.00.

The samples were measured in negative mode with a drying gas flow of 11 $L\cdot min^{-1}$ at 300 °C. The capillary voltage was set to 3000 V. The nebulizer pressure was 30 psi, and the fragmentor, skimmer, and octopole RF were set to 160 V, 65 V, and 400 V, respectively. Mass spectrometric detection was performed over an m/z range of 100–1100, with a scan rate of 1 $spectrum\cdot s^{-1}$.

III – LITERATURE FOR SUPPORTING INFORMATION

[1] White, R. H. Structural Characterization of Modified Folates in Archaea. Methods Enzymol. 1997, 281, 391−401. DOI: 10.1016/s0076-6879(97)81046-4

[2] Duin, E. C.; Prakash, D.; Brungess, C. In Methods in Enzymology; Rosenzweig, A. C.; Ragsdale, S. W., Eds.; Academic Press, 2011; pp 159−187. DOI: 10.1016/B978-0-12-385112-3.00009-3

[3] Breitung, J.; Börner, G.; Scholz, S.; Linder, D.; Stetter, K. O.; Thauer, R. K. Salt Dependence, Kinetic Properties and Catalytic Mechanism of N-Formylmethanofuran:Tetrahydromethanopterin Formyltransferase from the Extreme Thermophile Methanopyrus kandleri. Eur. J. Biochem. 1992, 210, 971−981. DOI: 10.1111/j.1432-1033.1992.tb17502.x

[4] Keltjens, J. T.; Caerteling, G. C.; Vogels, G. D. In Methods in Enzymology; Academic Press, 1986; pp 412−425. DOI: 10.1016/0076-6879(86)22201-6

[5] Croghan, W.; Egeghy, P. P. 2003. https://api.semanticscholar.org/CorpusID:12446551

[6] Cerdà, V.; Phansi, P.; Ferreira, S. From Mono- to Multicomponent Methods in UV−Vis Spectrophotometric and Fluorimetric Quantitative Analysis—A Review. TrAC Trends Anal. Chem. 2022, 157, 116772. DOI: 10.1016/j.trac.2022.116772
